# Evaluation of *Senna tora* (L.) Roxb. leaves as source of bioactive molecules with antioxidant, anti-inflammatory and antibacterial potential

**DOI:** 10.1016/j.heliyon.2023.e12855

**Published:** 2023-01-19

**Authors:** Md. Mashiar Rahman, Md. Abdullah Al Noman, Shapla Khatun, Rahat Alam, Md. Mahade Hasan Shetu, Enamul Kabir Talukder, Raihan Rahman Imon, Md. Yaman Biswas, K.M. Anis-Ul-Haque, Mohammad Jashim Uddin, Shahina Akhter

**Affiliations:** a Molecular and Cellular Biology Laboratory, Department of Genetic Engineering and Biotechnology, Jashore University of Science and Technology, Jashore 7408, Bangladesh; b Department of Chemistry, Jashore University of Science and Technology, Jashore 7408, Bangladesh; c Department of Pharmacy, Jashore University of Science and Technology, Jashore 7408, Bangladesh; d Department of Biochemistry and Biotechnology, University of Science and Technology Chittagong, Foy's Lake, Chittagong 4202, Bangladesh

**Keywords:** *Senna tora* (L.) Roxb., Antioxidant activity, Anti-inflammatory activity, Antibacterial activity, Phytochemicals, PASS, ADMET, Drug-able properties

## Abstract

*Senna tora* (L.) Roxb. is an ethno-medicinal herb used by rural and tribal people of the Satpura region of Madhya Pradesh in India and the Phatthalung Province of Thailand for treating rheumatism, bronchitis, ringworm, itches, leprosy, dyspepsia, liver disorders and heart disorders. It is also used in Chinese and Ayurvedic medicine. This study was conducted to investigate the potential of *Senna tora* (L.) Roxb. as a source of drug candidates against oxidants, inflammation, and bacterial infection. Preliminary phytochemical screening (PPS) and GC-MS were performed to identify the phytochemicals in the ethyl acetate extract of *Senna tora* (L.) Roxb. leaves (EAESTL). The *in vitro* antioxidant activity was assessed by 2,2-diphenyl-1-picrylhydrazyl (DPPH)- and H_2_O_2_-scavenging tests; the *in vitro* anti-inflammatory activity was determined by bovine serum albumin (BSA) denaturation and red blood cell (RBC) hemolysis inhibition; and the antibacterial activity was evaluated by agar-well diffusion methods. Cytotoxicity was estimated by *Artemia salina* larvae lethality, while acute toxicity was evaluated by oral delivery of the extract to mice. *In silico* antioxidant, anti-inflammatory, and antibacterial activities were predicted by the Prediction of Activity Spectra for Substances (PASS) program. The pharmacokinetics related to ADME and toxicity tests were determined by the admetSAR2 and ADMETlab2 web servers, and drug-able properties were assessed by the SwissADME server. GC-MS detected fifty-nine phytochemicals that support the types of compounds (phenols, flavonoids, tannins, terpenoids, saponins, steroids, alkaloids, glycosides and reducing sugar) identified by phytochemical screening. EAESTL exhibited dose-dependent antioxidant, anti-inflammatory, and antibacterial activities without any adverse effects or fluctuations in body weight. The PASS program predicted that the identified phytochemicals have antioxidant, anti-inflammatory and antibacterial activities. Among 51 phytochemicals, 16 showed good ADME, and 8 fulfilled drug-able properties without toxicity. Altogether, four phytochemicals, viz., benzyl alcohol, 3-(hydroxy-phenyl-methyl)-2,3-dimethyl-octan-4-one, phenylethyl alcohol and 2,6,6-trimethylbicyclo [3.1.1] heptane-3-ol, showed good pharmacokinetics and drug-able properties without toxicity, along with antioxidant, anti-inflammatory, and antibacterial activities. The obtained results suggest that *Senna tora* (L.) Roxb. leaves contain bioactive phytochemicals that have the potential to fight against oxidants, inflammation, and bacterial infection as potential drug candidates.

## Highlights

• The *Senna tora* leaves contained bioactive phytochemicals that had antioxidant, anti-inflammatory and antibacterial activities.
• Benzyl alcohol had antioxidant, anti-inflammatory, and antibacterial activity. After this in another bullet point write: 3-(hydroxy-phenyl-methyl)-2,3-dimethyl-octan-4-one had anti-inflammatory activity.
• Phenylethyl alcohol was found to have antioxidant and anti-inflammatory activity.
• 2,6,6-trimethylbicyclo [3.1.1] heptane-3-ol, also known as isopinocampheol/3-pinanol, could be an efficacious drug candidate against inflammation and bacterial infection.

## 1 Introduction

Humans receive medicinal plants as a gift from nature to aid in their quest for improved health. Since the dawn of humanity, mankind has recognized and utilized natural plant products as the prime source of therapeutic medications [1,2]. They are still a huge source of prominent and effective bioactive compounds that are capable of being employed as medications right away [2]. There are numerous examples of plant-based medications, like antimicrobial as nicotine, antioxidant as nordihydroguaiaretic acid (NDGA), anticancer as vinblastine, bleomycin, paclitaxin, and taxol, and anti-inflammatory as aescin and capsaicin, cardiotonic as acetyldigoxin, and antiparkinsonism as l-dopa, etc [1,3]. Their use is promoted in the Ayurvedic, Unani, Siddha, Homeopathic, and other frameworks of medication [1]. They pertain impact on huge number of human lives as customary medicines for different sicknesses like rheumatism, bronchitis, burns, itches, leprosy, colds, roundworm infection, wound bleeding, diarrhea and others [1,4]. Despite the discovery of numerous pharmaceuticals with plant origins, research into novel bioactive chemicals is still necessary to expand the available pharmacological options and look for less toxic medications with improved efficacy [5,6]. Therefore, the search for highly antioxidant, anti-inflammatory, and antimicrobial phytochemical compounds is one of the most intensive fields of research to minimize the risk of free radical-induced cellular damage or tissue injury, inflammation, bacterial infection, and associated diseases. Synthetic antioxidants are available for the treatment of oxidative stress-mediated oxidant-induced diseases and disorders but have associated health risks and severe adverse effects [7]. Nonsteroidal anti-inflammatory drugs (NSAIDs) are used for treating inflammation, but they have several detrimental consequences, including gastrointestinal ulcers, heartburn, kidney impairment, nausea, and vomiting [8,9]. Antimicrobials are losing their effectiveness, making it more difficult and occasionally impossible to cure diseases like pneumonia, TB, blood poisoning, gonorrhea, and foodborne diseases. New resistance mechanisms are emerging and spreading globally, threatening our ability to treat common infectious diseases [10]. In addition, the majority of currently available antibiotics have serious side effects, including life-threatening immune-related fallacious effects, anaphylaxis, dermatological cross-reactivity [11], hypersensitivity, and altering the body's normal microbiota, hence affecting normal physiological processes and making the lungs more vulnerable to viral diseases such as the flu [12]. The “back to nature” method has therefore gained significant acceptance in recent years. A large number of plant-based molecules have been developed and approved as antioxidants, anti-inflammatory agents and antibacterial potential therapeutics [[13], [14], [15]]. Hence, to find a feasible course of action that is safe and that lowers the adverse effects induced by chemotherapy, the continuous exploration of antioxidant, anti-inflammatory, and antibacterial phytochemicals plays a critical role.

*Senna tora* (L.) Roxb. Is a yearly foetid herb that belongs to the family Fabaceae and commonly propagates in barren land as a monsoon wild plant in humid countries (Bangladesh, India, Pakistan, and western China) [16–18]. Locally, it is recognized as chakramard in Ayurveda, chakunda/panevar/aranchi in Bengali, and foetid cassia/sickle pod/sickle senna in English [16–19]. It is also an ethno-medicinal herb used in traditional Chinese medicine and Ayurvedic medicine [[16], [17], [18],^20^]. Rural and tribal people and traditional healers use this plant to treat constipation, ulcers, ringworm, itch, appetizers, insomnia, liver disorders, leprosy, bronchitis, cough, dyspepsia, and heart diseases [[16], [17], [18],^20^]. The leaves have antirheumatic action in folklore preparation, and the leaf essence is used as a laxative [18]. Different parts of *Senna tora* (L.) Roxb. have been reported for their *in vitro* antioxidant and antibacterial activities and *in vivo* anti-inflammatory and analgesic activities due to the high content of different phytochemicals, such as tannins, saponins, reducing sugar, gum, steroids and alkaloids [21].

However, very little evidence related to *Senna tora* (L.) Roxb's phytochemical compounds is available, and the *in vivo* cytotoxicity and acute toxicity, pharmacokinetics (ADME), and drug-able properties have not been explored, though it is an important ethnomedicinal plant. No research has identified the specific bioactive phytochemicals in *Senna tora* (L.) Roxb. leaves that have biological activity against oxidants, inflammation, and bacterial infection and have good pharmacokinetic and drug-able properties without toxicity. Thus, this study focused on identifying the phytochemicals of the extract and assessing their *in vitro* antioxidant, anti-inflammatory, and antibacterial activities; evaluation of the cytotoxicity and acute toxicity; and prediction of the *in silico* probable biological activity, pharmacokinetics (ADME), *in silico* toxicity, and drug-able properties of identified phytochemicals extracted by ethyl acetate in an ideal drug development approach. The findings of this study will support further *in vivo* research into the potential therapeutic use of *Senna tora* (L.) Roxb. leaves against oxidants, inflammation, and bacterial infection, as well as the development of potential drugs for the treatment of associated diseases.

## 2 Materials and methods

### 2.1 Chemicals and reagents

BSA (CAS: 9048-46-8), DPPH (CAS: 1898-66-4), vincristine sulfate (CAS: 2068-78-2), and l-ascorbic acid (CAS: 50-81-7) were collected from Sigma-Aldrich, Chemie GmbH, Germany. Methanol (CAS: 67-56-1), ethyl acetate (CAS: 141-78-6), dimethyl sulfoxide (CAS: 67-68-5), ferric chloride (CAS: 10025-77-1), lead acetate (CAS: 6080-56-4), HCl (CAS: 7647-01-0), CuSO4 (CAS: 7758-99-8), H_2_SO_4_ (CAS: 7664-93-9), sodium nitroprusside (CAS: 13755-38-9), were purchased from Merck, Darmstadt, Germany. Pyridine (CAS: 110-86-1) and chloroform (CAS: 67-66-3) were from Alfa Aesar, Shore Road, Heysham, UK. NaCl (CAS: 7647-14-5), H_2_O_2_ (CAS: 7722-84-1) and NaOH (CAS: 1310-73-2) were purchased from Wako Pure Chemical Industries, Ltd., Japan. Mayer's solution and Fehling's solution were obtained from Central Drug House (P) Ltd., New Delhi, India. Nutrient broth and nutrient agar media were obtained from Liofilchem, Italy. Glacial acetic acid (CAS: 64-19-7) was acquired from PanReac AppliChem, Darmstadt, Germany. Erythromycin antibiotic discs were purchased from Bio-Rad, USA. Ibuprofen was gifted by Beximco Pharmaceuticals Ltd., Bangladesh.

### 2.2 Plant material

The *Senna tora* (L.) Roxb. leaves were taken in March 2019 from Churamonkathi, located at latitude 23.2238° N and longitude 89.1646° E in the Jashore District in Bangladesh. The plant material was botanically identified by Professor Dr. Shaikh Mizanur Rahman, Department of Genetic Engineering and Biotechnology, Jashore University of Science and Technology, Jashore-7408, Bangladesh. A voucher specimen was prepared and the plant's authentication was further confirmed by a taxonomist, Dr. Sardar Nasiruddin, at the National Herbarium, Dhaka, Bangladesh, and a voucher specimen (voucher number DACB 65146) was deposited at the National Herbarium, Dhaka, Bangladesh. The collected leaves were soaked and rinsed with water. An air-conditioned setting was utilized to dry the leaves at room temperature (∼25 °C). The leaves were dried and blended rigorously by a blender, and the obtained powder particles were stored in a sealed container.

### 2.3 Bacterial strains used

Both Gram-positive and Gram-negative bacteria were used in this study. From the Gram-positive category, *Bacillus infantis*, ATCC 15697 (*B. infantis*), *Exiguobacterium* sp., ATCC BAA-1283/AT1b (*Exiguobacterium* sp.), *Staphylococcus aureus* ATCC 23235 (*S. aureus*) and *Enterococcus* sp., ATCC 19952 (*Enterococcus* sp.) were tested in antibacterial experiments. *Escherichia coli* O157:H7 (*E. coli*), *Vibrio cholerae* O139 (*V. cholerae*), *Salmonella typhi* ATCC 13311 (*S. typhi*), *Pseudomonas aeruginosa* ATCC 27853 (*P. aeruginosa*), and *Haemophilus influenzae* ATCC 49247 (*H. influenzae*) represented the Gram-negative bacteria.

### 2.4 Experimental animals and ethical approval

The mice used in this study were collected from the Animal Center of Jahangirnagar University, Savar, Dhaka, Bangladesh. The mice were Swiss albino, male, 22–25 g in weight, and 6 weeks of age. The mice were accommodated in an animal house and the conditions were maintained in the animal house with a standard day-night (12-h) alternating light cycle at ambient temperature (23 ± 2 °C). Here, six mice were accommodated randomly in each of the polypropylene cages for the experiment. Free access to food and water, along with a normal supply, was arranged for the experimental mice. The animal experiment in this study was carried out in accordance with the ARRIVE guidelines [22]. The protocol (No. ERC/FBST/JUST/2021-91) was approved by the Institutional Ethical Committee of the Faculty of Biological Science of Jashore University of Science and Technology, Jashore-7408, Bangladesh. The use of human blood samples, such as the method of sample collection, privacy protection, data analysis, and decomposition plan, was validated by following the institutional standard.

### 2.5 Preparation of plant extract

The phytochemicals of *Senna tora* (L) Roxb. leaves were extracted by following the method explained previously with some small changes in the workup process [23]. The powdered plant product (500 g) was taken into five different conical flasks (1 L in size), and ethyl acetate (400 mL) was added to each flask. The conical flask was shaken for 72 h at 250 rpm by using a floor-standing shaking incubator (JSSI-300T, JSR, Republic of Korea), and the temperature was set at 37 °C. After this shaking, a few min of settling time were allowed, and clean cotton gauze was used to filter the mixture, followed by a second filtration by Whatman filter paper (no. 1). The sample was concentrated using a rotary evaporator (RE100-Pro, DLAB Scientific Inc., CA, USA), freeze-dried to obtain a powder, and stored in a sterile tube at 4 °C. The yield of extract from 500 g powder plant material was 20.55 g (dry weight, 4.11% w/w).

### 2.6 Qualitative phytochemical screening

The EAESTL solution was used in various qualitative analyses for phytochemicals. Alkaline reagent, FeCl_3_, and lead acetate tests were utilized to detect the existence of phenolic compounds (flavonoids and tannins). In an alkaline reagent test, a few drops of NaOH (5%) solution were added dropwise into the EAESTL solution (25 mg EAESTL dissolved in 2.5 mL methanol), which produced a deep-yellow color. Afterwards, a concentrated HCl (10%) solution was added to this alkaline solution and the color was lost just after adding the acid solution, which identified the presence of flavonoids [24]. To identify the presence of phenols, a few drops of FeCl_3_ (5%) solution were added to 2.0 mL of EAESTL solution (10 mg/mL) prepared in distilled water [25]. This test provides information regarding the presence of tannins in the EAESTL solution by developing a reddish-black color. A creamy-white color precipitation appeared after adding lead acetate solution (10%) to the 5 mg of EAESTL, indicating the availability of tannins. The Salkowski test was used to determine the presence of terpenoids and steroids [26]. In this test, 8 mg of EAESTL was shaken separately with 4 mL of chloroform, filtered to collect the clear part, and transferred to 2 test tubes evenly. Afterwards, H_2_SO_4_ (conc.) was added at the tube edge without shaking, and a bluish-brown layer at the interface suggested the presence of terpenoids. Concentrated H_2_SO_4_ was added to another tube of EAESTL and agitated; after settling, a black color appeared on the bottom face, indicating that steroids were present in the sample. In Mayer's test, 25 mg of EAESTL was dissolved in 10 mL of aqueous HCl (1%) with constant stirring in a water bath and then filtered off to collect the clear part. 1 mL of Mayer's reagent was mixed with 1.5 mL of this clear acid-treated EAESTL solution. A creamy-colored precipitate appeared at the edge, indicating the presence of alkaloids in EAESTL [25]. To detect cardiac glycosides, legal tests were utilized. 5 mg of EAESTL was treated with 3 mL of pyridine, and then 2 drops of nitroprusside and a drop of 20% sodium hydroxide solution were added to this solution. The appearance of a blood-red color demonstrated the existence of cardiac glycosides in EAESTL [24. Frothing and foaming tests were used to identify the presence of saponins in EAESTL [24]. Here, distilled water (3 mL) was added to 0.5 mg/mL of EAESTL in methanol and shaken for a few min before being allowed to stand. Even after 10 min of standing, the foam did not disappear, demonstrating the presence of saponins. In Fehling's test, 5 mg of EAESTL was added to an equal volume of Fehling's solutions A and B and boiled for a few min. Reducing sugar was identified by the sudden appearance of a yellowish color, which later turned into a brick-red precipitate [24].

### 2.7 Gas chromatography-mass spectrometry (GC-MS) analysis

GC-MS was conducted in accordance with a previously described method [27] with minor modifications. A gas chromatography-mass spectrometer (Shimadzu triple-quad GCMS-TQ8040) was used to identify the phytochemicals in EAESTL. Helium gas and an Rtx-5MS capillary column (30 m × 0.25 mm id, film thickness of 0.25 m) were utilized as the mobile and stationary phases, respectively. The column oven temperature was controlled by dedicated software, and graduated heating was set at 50, 200, and 300 °C at 1, 2, and 7 min, respectively. The temperature of the sample injector was 250 °C, with a 40-min experimental run time. This instrument was operated in splitless mode, where a 1 μL sample was injected. The constant flow rate was set at 1 mL/min. The detector parameters for GC–MS analyses were set as follows: 250 °C interface temperature, 230 °C ion source temperature, 50–600 (*m*/*z*) scanning range, and an ionization energy of 70 eV with a scan time of 0.3s. The phytochemical ingredients were identified by comparing the retention time and obtained pattern of the spectrum, which was then matched with the database of the National Institute of Standards and Technology (NIST). The individual peak area of an ingredient with respect to the total area of all other peaks was used to calculate the individual content (%) of an ingredient in EAESTL.

### 2.8 Antioxidant activity assay

#### 2.8.1 In vitro antioxidant activity assay by scavenging DPPH free radicals

DPPH was used to determine the free radical-scavenging activity of EAESTL by following a previously described method [24]. Individual stock solutions of DPPH (1.5 mg/mL), EAESTL (10 mg/mL), and ascorbic acid (10 mg/mL) were prepared in methanol. A DPPH solution (60 μL at 1.5 mg/mL) was added to different concentrations (200, 180, 160, 80, 40, 20, 10, 5, and 2.5 μg/mL) of EAESTL and ascorbic acid separately to a final concentration of DPPH 30 μg/mL and a final volume of 3.0 mL with methanol. The control was a 3.0 mL DPPH solution of 30 μg/mL concentration in methanol. The reaction mixtures were properly mixed and shaken, followed by 30 min of preservation in the dark. The absorbance was measured at 517 nm using a UV–vis spectrophotometer. For each analysis, three independent samples were prepared, and the average absorbance was used for the calculation. The free radical-scavenging activity was estimated utilizing the following formula (Eq. (1)):

(1) $$\%\;free\;radical–scavenging\;activity=\frac{\left(Abs_{\mathrm{control}}–Abs_{\mathrm{sample}}\;or\;Abs_{\mathrm{ascorbic}\;\mathrm{acid}}\right)}{Abs_{\mathrm{control}}}\times 100$$

where Abs_control_ and Abs_sample_ are the absorbance of the control and EAESTL-added samples, respectively. Abs_ascorbic acid_ represents the absorbance of the standard sample, where ascorbic acid was added instead of EAESTL. Microsoft (MS) Excel was used to determine the IC_50_ of EAESTL and ascorbic acid, where the percent inhibition was plotted with the respective concentration of the sample. The formula y = ax + b was used in the final calculation, where x is the IC_50_ and was calculated at the y = 50 position of the curve.

#### 2.8.2 Scavenging of hydrogen peroxide (H_2_O_2_)

The antioxidant activity of EAESTL was measured by H_2_O_2_-scavenging assay by following the method described earlier [28]. 1.0 M of H_2_O_2_ solution was prepared using phosphate-buffered saline (PBS, pH 7.4) and 10 mg/mL of EAESTL solution and 10 mg/mL of ascorbic acid solution were prepared in methanol as stock solutions. A total of 60 μL of a 1 M H_2_O_2_ solution was added to varying concentrations (200, 180, 160, 80, 40, 20, 10, 5, and 2.5 μg/mL) of EAESTL and ascorbic acid separately to obtain a final concentration of H_2_O_2_ of 20 mM and a final volume of 3.0 mL with 2880 μL of PBS and the rest of methanol. The control was 60 μL of 1 M H_2_O_2_ solution plus 2880 μL of PBS and the rest of the methanol for a final volume of 3.0 mL. Before measuring the absorbance data, the samples were kept for 10 min for settling purposes, and then the absorbance was recorded at a wavelength of 230 nm by utilizing a UV–vis spectrophotometer. The percent (%) H_2_O_2_-scavenging activity was estimated by the following equation (Eq. (2)):

(2) $$\%\;H_{2}O_{2}–scavenging\;activity=\frac{\left(Abs_{\mathrm{control}}–Abs_{\mathrm{sample}\;}or\;Abs_{\mathrm{ascorbic}\;\mathrm{acid}}\right)}{Abs_{\mathrm{control}}}\times 100$$

where Abs_control_ is the absorbance of the negative control sample, and Abs_sample_ is the absorbance of the sample, where EAESTL was added. Abs_ascorbic acid_ is the absorbance of the ascorbic acid-added sample. The IC_50_ values for EAESTL and ascorbic acid were calculated by following a method similar to that described above in Section 2.5, where the H_2_O_2_-scavenging activity was plotted against the concentration of the solution.

### 2.9 In vitro anti-inflammatory activity

#### 2.9.1 Heat-induced hemolysis of RBC assay

The RBC suspension solution (sparingly soluble) was prepared by following the previously explained method, with minor modifications [29]. The whole blood of a healthy human individual was collected in a heparinized centrifuge tube. After collection, the blood was centrifuged for 5 min at 3000 rpm and washed three times with an equal volume of normal saline (0.9% NaCl). The upper clear part was discarded from the top of the tube. The residue part was reconstituted as a 10% (v/v) RBC suspension with an isotonic sodium phosphate buffer solution (10 mM, pH 7.4). To prepare 1 L of a 10 mM sodium phosphate buffer solution, 1.379 g NaH_2_PO_4_, 1.419 g Na_2_HPO_4_ and 9.0 g NaCl were used. The stock solutions of EAESTL and ibuprofen were prepared by dissolving them separately into DMSO to a concentration of 10 mg/mL. The RBC suspension (0.05 mL) was added to the varying concentrations (200, 180, 160, 80, 40, 20, 10, 5, and 2.5 μg/mL) of EAESTL and ibuprofen separately to a final volume of 3.0 mL with an isotonic sodium phosphate buffer solution. The control samples contained 0.05 mL of RBC suspension plus the same amount of DMSO instead of the tested sample and an isotonic sodium phosphate buffer solution with a final volume of 3.0 mL. In a shaking water bath, these samples were incubated at 54 °C for 20 min with constant shaking. After centrifugation at 2500 rpm, the clear part was collected from the top of the centrifuge tube. The absorbance of these sample solutions was measured at a 540 nm wavelength by using a UV–vis spectrophotometer. The following equation (Eq. (3)) was used to estimate the percent inhibition of hemolysis:

(3) $$\%\;inhibition=\left(1-\frac{Abs_{\mathrm{sample}}}{Abs_{\mathrm{control}}}\right)\times 100$$

Here, Abs_sample_ and Abs_control_ are the absorbances in the presence and absence of the EAESTL sample in PBS, respectively. Similarly, the ibuprofen-added sample was also compared with the control sample. The IC_50_ value was calculated by following a similar method described in Section 2.5, where the percent inhibition of erythrocyte hemolysis was plotted against the concentration of the respective sample.

#### 2.9.2 BSA protein denaturation assay

This experiment was performed by following a previously reported method with a slight modification [29]. 0.12 mL of 1% BSA was added to the varying concentrations (200, 180, 160, 80, 40, 20, 10, 5, and 2.5 g/mL) of EAESTL and ibuprofen separately to obtain a final concentration of BSA 0.04% and a final volume of 3.0 mL with phosphate buffered saline (PBS 1×, pH 7.4). The control samples were prepared by mixing 0.12 mL of 1% BSA with the same amount of DMSO instead of the tested sample and 1× phosphate buffered saline to a final volume of 3.0 mL and a final concentration of 0.04% BSA. All the samples were incubated in a water bath for 15 min at 37 °C, followed by an additional 5 min at 70 °C. The samples were allowed to cool, and the absorbance was recorded at a wavelength of 660 nm utilizing a UV–vis spectrophotometer. The following equation (Eq. (4)) was used to estimate the percent inhibition of protein denaturation:

(4) $$\%\;inhibition=\frac{\left({Abs}_{Control}-{Abs}_{Test\;sample}\right)}{{Abs}_{Control}}\times 100$$

Here, Abs_control_ and Abs_Test sample_ are the absorbance of the negative control and EAESTL or ibuprofen added sample at a wavelength of 660 nm, respectively. The IC_50_ of EAESTL and standard (ibuprofen) sample was calculated by following the same method described above in Section 2.5, where the percent inhibition of BSA denaturation was plotted against the concentration of the test sample.

### 2.10 Antibacterial activity assay

#### 2.10.1 Agar well diffusion assay

The agar well diffusion technique [24] was used to evaluate the antibacterial activity of EAESTL. All the tested bacterial strains were sub-cultured first by the streak plate technique on nutrient agar petri-plates. A single colony was taken from each bacterial culture plate of an individual strain and incubated in 25 mL of nutrient broth medium at 37 °C in a shaking incubator at 250 rpm and cultured to the mid-log phase of absorbance at 0.4 at 600 nm wavelength. 100 μL of fresh mid-log phase bacterial culture of each strain was spread on an individual nutrient agar petri-plate by a glass spreader and dried for 20 min. The four wells were produced by utilizing a 6 mm sterile cork borer on each nutrient agar petri-plate containing bacterial inoculums. A stock solution of EAESTL (500 mg/mL) was dissolved in DMSO and serially diluted to prepare 250, 125, 62.5 mg/mL concentrations of EAESTL. 100 μL of EAESTL solution of an individual concentration was applied sequentially in an individual well of the nutrient agar petri-plate. As a positive control, an antibiotic disc (erythromycin) was placed in the middle of the nutrient agar petri plate. As a negative control, 100 μL of DMSO was also applied into the well of nutrient agar petri-plate containing bacterial inoculums. Then, the nutrient agar petri-plates were kept in a refrigerator at 4 °C for 30 min to allow the samples to diffuse properly in the nutrient agar medium, followed by incubation at 37 °C for 16 h. The antibacterial activity was estimated by the zone of inhibition (within a well diameter) on the agar plate. The antibacterial activity assay was performed in triplicate.

#### 2.10.2 Minimum inhibitory concentration and minimum bactericidal concentration determination

The twofold serial dilution method was used to test the minimum inhibitory concentration (MIC) and minimum bactericidal concentration (MBC) of EAESTL [30]. A 500 mg/mL stock solution was placed in an autoclavable 15-mL glass tube and serially diluted with LB broth to achieve 250, 125, 62.5, 31.25, 15.625, 7.812, 3.906, and 1.953 mg/mL. A 50 μL bacterial culture in mid-log phase (absorption at 600 nm reached 0.4) was transferred to each tube. The control tube contained only bacterial strains and not the plant extract. The culture tubes were incubated at 37 °C for 24 h, and the turbidity of the tubes was examined. The lowest concentration of EAESTL that inhibited visible bacterial growth was considered the MIC. To determine the minimum bactericidal concentration of EAESTL, 50 μL of each culture was subcultured on the surface of an LB agar plate at 37 °C for 16 h. MBC was recorded as the lowest concentration of EAESTL that did not permit any visible bacterial colony growth on the LB agar plate after the incubation period. The MIC and MBC assays were performed three times.

### 2.11 Test of cytotoxicity by *Artemia salina* larvae lethality bioassay

The cytotoxicity of EAESTL was assessed using *Artemia salina* larvae as a test organism [31]. *Artemia salina* eggs were purchased from an aquarium shop in Dhaka, Bangladesh, and artificial seawater was used for hatching purposes. To prepare the artificial seawater, 38 g NaCl salt was dissolved in 1 L water followed by 48 h of hatching at 25 °C with constant aeration and irradiation of light. Active *Artemia salina* larvae were taken from this hatching and shifted to another vessel that contained fresh artificial seawater. The EAESTL and vincristine sulfate were dissolved separately into DMSO to a final concentration of 10 mg/mL. Each 0.1 mL of these solutions was mixed separately with 9.9 mL of artificial seawater in a total volume of 10 mL of 100 μg/mL concentration, from which serially diluted solutions were made to obtain a series of concentrations of 50, 25, 12.5, 6.25, and 3.125 μg/mL. An identical control was prepared without adding test samples but by diluting 0.05 mL of DMSO in 4.95 mL of artificial seawater to a final concentration of 1% DMSO, which is the DMSO concentration used in test samples. Ten (10) *Artemia salina* larvae per vial that contained either test or negative control samples were accommodated and examined after 24 h using a magnifying glass. The data were recorded based on the number of surviving *Artemia salina* larvae per vial. The following equation (Eq. (5)) was utilized for the estimation of the mortality of *Artemia salina* larvae for each concentration:

(5) $$\%\;mortality=\frac{\left(N_{T}-N_{L}\right)}{N_{T}}\times 100$$

where N_T_ and N_L_ are the quantity of *Artemia salina* larvae taken and alive, respectively, after a 1-day incubation period. The LC_50_ was quantified using a mortality-versus-concentration curve, where the LC_50_ value was calculated at y = 50 by following a method similar to that described for IC_50_ in Section 2.5.

### 2.12 Acute oral toxicity study

The Organization for Economic Co-operation and Development (OECD) (Test No. 425) was followed during the assessment of the acute toxicity of EAESTL on Swiss albino mice [32]. An acute oral toxicity study was performed according to the method described previously [24]. Forty-two (42) male mice were divided into seven groups (6 in each), namely, the g-250, g-500, g-1000, g-2000, g-4000, g-5000, and control groups. Before starting the experiment, the mice were starved for 3–4 h, and only water was allowed during this time. A stock solution of EAESTL (500 mg/mL) was dissolved in 2% DMSO in a 0.9% saline solution. Each mouse in the g-250, g-500, g-1000, g-2000, g-4000, and g-5000 groups received a single oral dosage that contained 250, 500, 1000, 2000, 4000, and 5000 mg/kg EAESTL, respectively. A blank dosage containing only 0.25 mL DMSO (2% in 0.9% saline) was given to each of the mice in the control group. Following treatment, mice were allowed to feed ad libitum and were monitored for the development of any symptoms of toxicity (vomiting, diarrhea, ataxia, and death) after dosage administration. These monitoring data were recorded after 3 h of dosage administration and then every 4 h for 1 day. For the remaining 13 days, data was recorded once a day. The mortality of mice was calculated after 48 h of oral dosage administration using the following equation (Eq. (6)):

(6) $$\%\;mortality=\frac{\left(N_{MT}-N_{ML}\right)}{N_{MT}}\times 100$$

where NMT and NML are the number of mice taken at the beginning and alive 48 h after EAESTL administration, respectively. The LD_50_ was calculated by following a similar method described above in Section 2.5. Here, the mortality was plotted with respect to the dose amount (concentration) taken in the relevant test sample. At the end of the experiment, mice were placed in a new cage with corn cob bedding and immediately euthanized by displacement of air with 100% carbon dioxide, followed by being burial in soil at an approved landfill at our university. This experiment was repeated three times.

### 2.13 Bodyweight measurement

The body weight of the selected mice (six mice in each group) groups (g-Control, g-250, g-500, g-1000, and g-2000) was measured by following a reported method [33]. In summary, the initial body weight was recorded just before the extract was administered. The body weight of the mice was monitored for 2 weeks and weighed after 3, 6, 9, 12, and 14 days of extract administration. The cumulative weight change was estimated by using the following equation (Eq. (7)):

(7) $$\%\;cumulative\;weight\;change=\frac{\left({CW}_{INTV}-{CW}_{\mathrm{INT}}\right)}{{CW}_{INTV}}\times 100$$

where CW_INTV_ represents the cumulative weight for a particular group at a specific time and CW_INT_ is the same parameter at the initial time of the experiment.

### 2.14 Computer-aided prediction of the biological activity of phytochemicals

The PASS online server assessed the possible biological functions of EAESTL phytochemicals in the context of biological activities [34]. Briefly, the name of each phytochemical was submitted to the PubChem server, and a canonical SMILES of this element was extracted into the PASS program (http://www.way2drug.com/PASSOnline/) to obtain the probable biological activity of a specific phytochemical. The outcome was obtained as a ratio of ‘probability of being active (Pa)’ and ‘probability of being inactive (Pi)’ in the context of Pa>0.3. A higher Pa for a phytochemical indicates a greater chance of having a particular pattern of biological activity.

### 2.15 Prediction of pharmacokinetics (ADME), toxicity and drug-able properties

Different properties of the identified phytochemicals were evaluated in terms of being ideal drug candidates. The pharmacokinetic properties were related to ADME (absorption, distribution, metabolism, and excretion), toxicity (hERG inhibitor, AMES toxicity, carcinogenicity, hepatotoxicity, and rat oral acute toxicity), and drug-able properties [physicochemical parameters (MW, HBAs, HBD, RTB, and TPSA), lipophilicity (cLogP), water solubility (LogS), drug-likeness (Lipinski's Rule of Five: RO5) and medicinal chemistry (synthetic accessibility)]. The AdmetSAR2 web server (http://lmmd.ecust.edu.cn/admetsar2/) [35] was utilized to anticipate absorption (human intestinal absorption = HIA, human oral bioavailability = HOB, p-glycoprotein inhibitors, p-glycoprotein substrates), distribution (BBB penetration), metabolism (CYP450 inhibitors and CYP450 substrates), and toxicity (hepatotoxicity). The canonical SMILES ID of each phytochemical was submitted into the admetSAR2 web server, and the result was recorded for a set of parameters. Plasma protein binding (PPB) (distribution), excretion (renal clearance), and toxicity were predicted by the ADMETlab2 server (https://admetmesh.scbdd.com/) [36]. To do this, the canonical SMILES ID of each phytochemical ingredient was submitted to the ADMETlab2 server, and the result was obtained for a selected set of parameters. However, the SMILES format data for identified phytochemicals were further uploaded to the SwissADME server (http://www.swissadme.ch/), and computational results for physicochemical properties, lipophilicity, water solubility, drug-likeness and a synthetic accessibility score were collected [37].

### 2.16 Statistical analysis

The experimental data are represented as the average ± STD of the replicates for all experiments except body weight measurements of mice after oral administration of different concentrations of extract. The statistical interpretation of tests was accomplished using one-way ANOVA by Origin Lab version 2018, and Bonferroni's and Tukey's post hoc tests were used for the tests where *p < 0.05 and **p < 0.01 are significant compared with control values.

## 3 Results

### 3.1 Phytochemical analysis of ethyl acetate extract of *Senna tora* (L.) Roxb. leaves (EAESTL)

Phytochemicals are indispensable in plant defense and have been used as important raw materials for pharmaceutical manufacturing for centuries. Therefore, we tested the existence of phytochemicals in the ethyl acetate extract of *Senna tora* (L.) Roxb. leaves (EAESTL) (Supplementary Figure S1). EAESTL was found to contain phenols (flavonoids and tannins), terpenoids, saponins, steroids, cardiac glycosides and reducing sugars, as listed in Table 1 and Supplementary Figure S2.

**Table 1:** Preliminary phytochemical screening of the ethyl acetate extract of *Senna tora* (L.) Roxb. leaves.

Test name	Observation	Result	Name of phytochemicals
Alkaline reagent	Colorless solution	+	Phenolic (Flavonoids)
Ferric chloride	Reddish-black coloration	+	Phenolic (Tannins)
Lead acetate	Creamy-white color precipitation	+	Phenolic (Tannins)
Salkowski's	Bluish-brown layer at upper interface	+	Terpenoids
Salkowski's	Black color in the bottom face	+	Steroids
Mayer's	Creamy-colored precipitation	+	Alkaloids
Legal's	Blood-red color	+	Cardiac glycosides
Foaming	Foam formation	+	Saponins
Fehling's	Brick-red precipitation	+	Reducing sugar

“+” means presence of specific phytochemicals.

### 3.2 GC-MS analysis of phytochemicals and predicting their antioxidant, anti-inflammatory, and antibacterial properties using PASS program

EAESTL had 59 peaks in its GC–MS chromatogram (Supplementary Figure S3). Each peak represented the existence of individual phytochemicals based on retention time (RT), peak area (%), chemical formula, compound structure and *in silico*, *in vitro* and/or *in vivo* biological properties (Table 2). The major phytochemicals identified in EAESTL were esters [methyl hexadecanoate (PN53 in Table 2, 7.28%), 2-(2-(2-butoxyethoxy)ethoxy)ethyl 2-methylbutanoate (PN40, 3.65%), isopentyl acetate (PN34, 3.35%), methyl stearate (PN55, 1.34%), trimethylsilyl 3-methyl-4-[(trimethylsilyl)oxy] benzoate (PN15, 1.33%) and decyl (2-methylpentyl) fumarate (PN22, 1.03%)], phenolics [phenylethyl alcohol (PN37, 3.65%), resorcinol (PN43, 3.58%), benzyl alcohol (PN28, 2.78%) and 4-(methoxymethyl)phenol, (PN4, 2.11%)], aldehyde [3-furaldehyde (PN5, 4.82%), 5-(hydroxymethyl)furan-2-carbaldehyde (PN42, 4.03%), and 5-methylfuran-2-carbaldehyde (PN17, 2.76%)], silicon compounds [octamethyl cyclotetrasiloxane, (PN39, 3.49%), decamethyl cyclopentasiloxane, (PN38, 2.26%), octamethylcyclotetrasiloxane (PN20, 2.10%), dimethyl 2,2'-((1,1,3,3,5,5-hexamethyltrisiloxane-1,5-diyl)bis(oxy))diacetate (PN27, 1.94%) and 1,1,3,3,5,5,7,7,9,9-decamethyl-1,9-pentasiloxane (PN44, 1.03%)], terpenoids [neophytadiene (PN50, 4.17%), (2E,7R,11R)-3,7,11,15-tetramethyl-2-hexadecen-1-ol (PN52, 2.05%) and 3,7,11,15-tetramethyl-2-hexadecen-1-ol (PN51, 1.46%)], alkaloids [phenyl carbamate (PN19, 2.44%) and 2,3,4,6,7,8-hexahydropyrrolo[1,2-a]pyrimidine (32, 1.13%)], alcoholic compounds [*tert*-butyldimethyl((3-methylbenzyl)oxy)silane (PN8, 1.97%) and benzyl alcohol (PN28, 2.78%)], silyl ether [benzyl(benzyloxy) dimethylsilane (PN10, 1.20%)], cyclic amino acid [piperidine-2-carboxylic acid (PN1, 2.17%)], omega-3 fatty acid [methyl (8E,11E,14E)-docosa-8,11,14-trienoate (PN55, 1.88%)], alkane [tetradecane (PN47, 1.44%)], organosulfur [2-methyl-3-(methylthio)furan (PN29, 1.47%)], steroids [stigmasta-5,22-dien-3beta-ol, acetate (PN57, 1.34%)], organic peroxide [3-hydroperoxyhexane (PN18, 1.25%)] and an aromatic compound [7a-methyloctahydro-2,7,4-(epiethane [1,1,2]triyl)cyclopenta[b]pyran (PN16, 1.15%)]. The relative content of phytochemicals in EAESTL was determined based on the peak area (%), and the order of occurrence was esters > phenolics > aldehydes > silicon compounds > terpenoids > alkaloids > heterocyclic compounds > alcoholic compounds > silyl ethers > cyclic amino acids > omega-3 fatty acids > alkanes > organosulfur > steroids > organic peroxides > aromatic compounds. The biological activity of a phytoconstituent is mostly inherent in the chemical and structural nature of the compound. Applications of medicinal plants are generally based on their biological activity, which is a prerequisite for consideration as a drug candidate. PASS program analysis of 59 phytochemicals predicted that 51 of them have pharmacological activities in the context of antioxidant, anti-inflammatory, and antibacterial activities. However, eight phytochemicals [hexamethylcyclotrisiloxane (PN2), 3-furaldehyde (PN5), TBDMS derivative (PN8), 4,2,7-ethanylylidenecyclopenta[b]pyran (PN16), phenyl carbamate (PN19), 2-methyl-3-(methylthio) furan (PN29), N-isobutylbicyclo [2.2.1] heptane-2-carboxamide (PN33), and pentasiloxane, 1,1,3,3,5,5,7,7,9,9-decamethyl (PN44)] did not have any antioxidant, anti-inflammatory, or antibacterial activities. Among fifty-one phytochemicals, thirty (PN3, PN4, PN6, PN7, PN11, PN12, PN15, PN18, PN20, PN22, PN23, PN24, PN27, PN28, PN32, PN34, PN35, PN37, PN38, PN39, PN40, PN41, PN45, PN46, PN47, PN49, PN53, PN54, PN56, and PN57) showed only anti-inflammatory activity, thirteen (PN1, PN9, PN10, PN13, PN14, PN17, PN21, PN26, PN31, PN36, PN42, PN51, and PN55) exhibited dual activities (anti-inflammatory and antibacterial), three (PN43, PN52 and PN59) showed triple activities (anti-inflammatory, antibacterial and antioxidant), two (PN30 and PN50) showed only antibacterial activity, one (PN25) exerted antibacterial and antioxidant activities, and two (PN48 and PN58) showed anti-inflammatory and antioxidant activities. This finding suggests that the presence of these phytochemicals in the extract is responsible for the *in vitro* antioxidant, anti-inflammatory, and antibacterial properties that we have shown in this study.

**Table 2:** GC–MS identified phytochemicals in the ethyl acetate extract of *Senna tora* (L.) Roxb. leaves. The phytochemical functionalities and their corresponding probable biological activities (PBAs) were predicted using the PASS program.

Peak No	Retention time	Area %	Structure, name and formula of the compound	Nature of compound	Compound CID	*In silico* PBA	Pa > 0,3		*In vitro* and/or *in vivo* biological activity
							Pa	Pi	
1	3.620	2.17		Cyclic amino acid	849	AI	0, 357	0, 038	Antibacterial [38] and inhibitor against EAC tumor cells [39]
						ABO	0,380	0, 003	
2	3.674	0.52		Organosilicon	10914	–	–	–	Antimicrobial & antioxidant [40] and antidiabetic [41]
3	3.741	0.70		Diol/dihydric alcohol	250594	A	0, 426	0, 083	No activity reported
						AI	0, 409	0, 020	
						AO	0, 329	0, 044	
4	3.815	2.11		Phenol	79310	A	0, 345	0, 126	Antibacterial [42] and neuroprotective [43]
						AI	0, 573	0, 004	
						AO	0, 375	0, 017	
5	3.905	4.82		Aldehyde	10351	–	–	–	Nrf2-keap1 pathway activator in skin cells [44]
6	3.951	0.67		Ketone	70258	A	0, 367	0, 026	Photosynthesis inhibitor [45] and anticancer (breast) [46]
						AI	0, 461	0, 011	
						AO	0, 420	0, 008	
7	3.998	0.51		Esters	91698641	A	0, 400	0, 095	No activity reported
						AI	0, 321	0, 056	
						AO	0, 470	0, 005	
8	4.040	1.97		Silyl ether	22967275	–	–	–	No activity reported
9	4.075	0.88		Unsaturated aldehyde	5283345	A	0, 391	0, 101	Antifungal [47] and antioxidant [48]
						AI	0, 427	0, 017	
						AB	0, 334	0, 048	
10	4.195	1.20		Silyl ether	518982	A	0, 391	0, 101	No activity reported
						AI	0, 427	0, 017	
						AB	0, 334	0, 048	
11	4.275	0.68		Aromatic hydrocarbon	7237	A	0, 375	0, 109	Antibacterial [49]
						AI	0, 459	0, 011	
12	4.490	0.72		Polyunsaturated hydrocarbon	637866	A	0, 315	0, 147	No activity reported
						AI	0, 519	0, 006	
						AO	0, 399	0, 010	
13	4.685	0.75		Heterocyclic organic compound	10341	A	0, 434	0, 079	Antioxidant, anti-inflammatory, analgesic, antibacterial anticancer, anticonvulsant, antifungal, antiulcer and anti-TB [50]
						AI	0, 359	0, 036	
						AO	0, 326	0, 047	
						AB	0, 414	0, 027	
14	4.805	0.57		Monoterpene	99038	AO	0, 331	0, 042	Antifungal [51]
						AB	0, 398	0, 030	
15	5.150	1.33		Ester	91740684	AI	0, 344	0, 043	Protects human cells against oxidative damage and cancer [52]
16	5.215	1.15		Aromatic compound	565150	–	–	–	No activity reported
17	5.306	2.76		Aldehyde	12097	AI	0, 306	0, 066	Antioxidant [53]
						AB	0, 417	0, 026	
18	5.360	1.25		Organic Peroxide	141085	AI	0, 437	0, 015	No activity reported
						AO	0, 345	0, 032	
19	5.441	2.44		Alkaloids	69322	–	–	–	Acetylcholinesterase inhibitor [54] and antifungal [55]
20	5.485	3.49		Organosilicon	11169	AI	0, 342	0, 044	Estrogenic [56]
21	5.590	0.97		Esters	137020	A	0, 765	0, 009	No activity reported
						AO	0, 355	0, 025	
						AB	0, 427	0, 025	
22	5.622	1.03		Esters	91737497	A	0, 515	0, 053	No activity reported
						AI	0, 343	0, 044	
23	5.650	0.99		Alcoholic compound	542357	AI	0, 377	0, 030	No activity reported
						AO	0, 407	0, 009	
24	5.725	1.39		Bromine compound	98219	AI	0, 402	0, 022	Antimicrobial [57]
25	5.755	0.58		Vitamin-C	54685836	AOx	0, 381	0, 014	No activity reported
						AB	0, 381	0, 035	
26	6.073	0.80		Haloalkane	33574	AI	0, 425	0, 017	No activity reported
						AB	0, 327	0, 050	
27	6.109	1.94		Organosilicon	91738767	AI	0, 320	0, 057	No activity reported
28	6.166	2.78		Aromatic alcohol/Pehnol	244	A	0, 414	0, 089	Analgesic [58], antioxidant and antibacterial [59]
						AI	0, 532	0, 005	
						AO	0, 363	0, 021	
29	6.282	1.47		Aryl thioethers (organosulfur)	526618	–	–	–	No activity reported
30	6.340	0.50		Ester	607122	AB	0, 346	0, 044	No activity reported
31	6.570	0.53		Fatty aldehyde	5364438	AI	0, 427	0, 017	Mosquito larvicidal [60]
						AB	0, 400	0, 030	
32	6.76	1.13		Amidine base/Alkaloids	76349	A	0, 515	0, 052	No activity reported
33	6.81	3.6		Carboxamide (Amide)	565668	–	–	–	No activity reported
34	6.9	3.35		Ester	31276	A	0, 602	0, 031	No activity reported
						AI	0, 323	0, 055	
						AO	0, 354	0, 026	
35	7.075	0.49		Aromatic alcohol/Phenol	559104	A	0, 415	0, 053	No activity reported
						AI	0, 570	0, 004	
						AO	0, 305	0, 072	
36	7.105	0.63		Unsaturated heterocyclic ketone	699486	A	0, 875	0, 005	No activity reported
						AI	0, 410	0, 020	
						AO	0, 450	0, 005	
						AB	0, 353	0, 042	
37	8.025	3.65		Aromatic alcohol/Phenol	6054	AO	0,450	0,005	Antioxidant [61], antifungal [62] and anti-inflammatory [63]
						AI	0,410	0,020	
						A	0,305	0,156	
38	7.218	2.26		Organosilicon	10913	AI	0, 342	0, 044	No activity reported
39	7.946	2.10		Silicon compound	6453921	AI	0, 342	0, 044	Antioxidant and antimicrobial [64]
40	8.025	3.65		Ester	91693497	A	0, 531	0, 048	No activity reported
						AI	0, 311	0, 062	
						AO	0,372	0, 018	
41	8.185	3.90		Heterocyclic Compound	10329	A	0, 521	0, 051	Antileishmanial [65]
						AI	0, 353	0, 039	
						AO	0, 359	0, 023	
42	8.248	4.03		Aromatic aldehyde	237332	AI	0, 328	0, 052	Anti-sickling [66], antioxidant and antiproliferative [67]
						AB	0, 407	0, 028	
43	8.793	3.58		Phenol	5054	AOx	0, 486	0, 007	Antioxidative [68] and anticancer [69]
						A	0, 496	0, 058	
						AI	0, 537	0, 005	
						AO	0, 369	0, 019	
						AB	0, 310	0, 057	
44	8.958	1.03		Organosilicon	6329089	–	–	–	No activity reported
45	9.008	0.88		Organosilicon	10911	AI	0, 342	0, 044	No activity reported
46	9.287	0.52		Oxo alcohol	24847	A	0, 543	0, 045	No activity reported
						AI	0, 440	0, 014	
						AO	0, 356	0, 025	
47	10.059	1.44		Long chain alkane	12389	A	0, 424	0, 084	Antibacterial and antifungal [70]
						AI	0, 585	0, 004	
						AO	0, 378	0, 015	
48	11.075	0.48		Aromatic alcohol/Phenol	70825	AOx	0, 469	0, 008	Antifungal [71]
						A	0, 610	0, 029	
						AI	0, 449	0, 013	
						AO	0, 313	0, 061	
49	11.075	0.52		Hydrocarbon	12391	A	0, 424	0, 084	No activity reported
						AI	0, 585	0, 004	
						AO	0, 378	0, 015	
50	14.898	4.17		Terpenoid	10446	AB	0, 363	0, 040	Antimicrobial and antioxidant [72]
51	15.241	1.46		Diterpene	5366244	A	0, 458	0, 070	Anticarcinogenic and antitumor [73], antimicrobial, anti-inflammatory, anticancer and antiartrhitis [74] and antinociceptive and antioxidant [75]
						AI	0, 305	0, 067	
						AB	0, 417	0, 026	
52	15.529	2.05		Diterpene	5280435	AOx	0, 475	0, 008	Antinociceptive and antioxidant [75]
						A	0, 458	0, 070	
						AI	0, 305	0, 067	
						AB	0, 417	0, 026	
53	16.184	7.28		Fatty acid methyl ester	8181	A	0, 510	0, 054	Anti-inflammatory [76]
						AI	0, 758	0, 002	
						AO	0, 441	0, 005	
54	18.905	0.48		Ester	534619	A	0, 330	0, 135	No activity reported
						AI	0, 380	0, 029	
						AO	0, 446	0, 005	
55	19.008	1.88		Omega 3 fatty acid	5364473	A	0, 728	0, 013	No activity reported
						AI	0, 681	0, 003	
						AO	0, 407	0, 009	
						AB	0, 302	0, 059	
56	19.435	1.34		Fatty acid ester	8201	A	0, 510	0, 054	Antibacterial, antioxidant and antitumor [77] and anticancer [78]
						AI	0, 758	0, 002	
						AO	0, 441	0, 005	
57	33.568	1.34		Steroid	6432445	A	0, 546	0, 044	Antifungal [79]
						AO	0, 402	0, 010	
58	34.901	0.65		Synthetic form of vitamin E	2116	AOx	0, 967	0, 002	Anticancer [80], anti-inflammatory, antioxidant, antimicrobial, radical scavenging and antispasmodic [81]
						A	0, 814	0, 006	
						AO	0, 587	0, 003	
59	37.218	0.58		Carotene	5366411	AOx	0, 456	0, 008	Antioxidant, antiasthma, anticancer, immune enhancement, arthrosclerosis and gingivitis prevention [82]
						A	0, 561	0, 040	
						AI	0, 378	0, 029	
						AB	0, 336	0, 047	

Compound's probable biological activity (PBA) was determined in the aspect of only antioxidant, anti-inflammatory, and antibacterial activity and Pa>0.3. Here, AOx = Antioxidant, A = Anti-inflammatory, AI = Anti-inflammatory intestinal, AO = Anti-inflammatory ophthalmic, AB = Anti-bacterial, ABO = Antibacterial ophthalmic.

### 3.3 DPPH and hydrogen peroxide radical scavenging activity

**Fig. 1:**
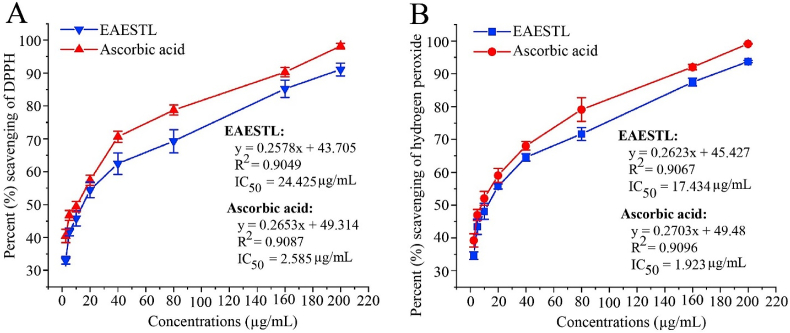
The antioxidant activity of EAESTL was compared with that of ascorbic acid. (A) DPPH free radical scavenging by EAESTL and ascorbic acid. (B) The H_2_O_2_-scavenging activity of EAESTL and ascorbic acid. The mean standard deviation (STD) of three individual tests was employed to calculate the results.

DPPH is a nitrogen-extractable oxidant that is conventionally used to determine the free radical-scavenging activities of antioxidants present in plant extracts or synthetic compounds. EAESTL at concentrations of 2.5, 5, 10, 20, 40, 80, 160, and 200 μg/mL exhibited dose-dependent antioxidant activity (Fig. 1A), with a range from 33.041 ± 1.166% to 91.068 ± 1.950% DPPH inhibition and an IC_50_ value of 24.425 μg/mL. Similar antioxidant activity was shown by ascorbic acid, ranging from 40.455 ± 2.019% to 98.190 ± 0.863% DPPH restraint with an IC_50_ value of 2.585 μg/mL. Although H_2_O_2_ is not a free radical, it plays a pivotal role in causing oxidative damage by transforming into a highly reactive hydroxyl radical (HO^•^). Therefore, we also performed an H_2_O_2_-scavenging assay for EAESTL and ascorbic acid. This analysis found concentration-dependent antioxidant activity for EAESTL by scavenging H_2_O_2_ with a range of 34.595 ± 1.104% to 93.734 ± 0.336% and an IC_50_ value of 17.434 μg/mL (Fig. 1B). Moreover, ascorbic acid showed H_2_O_2_-scavenging activity comparable with that of EAESTL, and the range was 39.238 ± 2.040 to 99.154 ± 0.115% with an IC_50_ value of 1.923 μg/mL. In summary, comparing the results obtained in different scavenging assays, EAESTL and a well-known antioxidant (ascorbic acid) showed similar antioxidant activity.

### 3.4 EAESTL exhibited potent anti-inflammatory activity

**Fig. 2:**
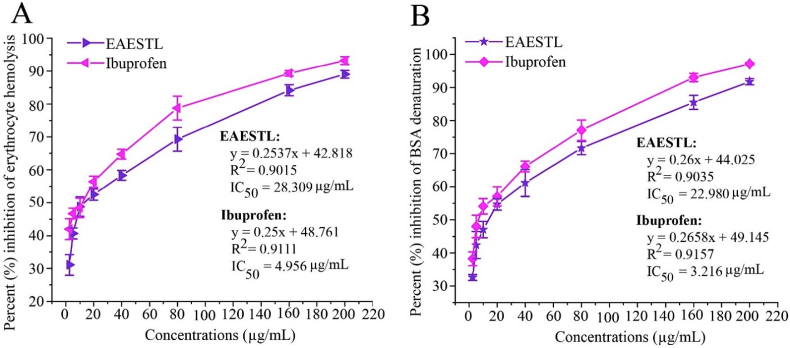
The anti-inflammatory activity of EAESTL was contrasted with ibuprofen. The anti-inflammatory activity of EAESTL was ascertained by the heat-induced hemolysis of RBC repression (A) and BSA distortion inhibition (B). The mean ± STD of three individual tests was used to analyze the outcomes.

Inflammation is firmly related to pathophysiological processes. The quest for natural compounds and phytoconstituents that might prevent inflammation could be helpful for human health. In this study, EAESTL displayed dose-related anti-inflammatory activity by inhibiting RBC hemolysis and BSA denaturation. At different concentrations, EAESTL inhibited hemolysis of the RBC membrane within a range of 31.058 ± 3.145% to 89.029 ± 1.186%, where the IC_50_ value was 28.309 μg/mL (Fig. 2A). In addition, the BSA denaturation inhibition of EAESTL ranged from 32.617 ± 0.890% to 91.731 ± 0.949, and the IC_50_ value was 22.980 μg/mL (Fig. 2B). The anti-inflammatory activity of EAESTL was compared with ibuprofen, which commonly functions as an active ingredient in standard anti-inflammatory drugs. It showed anti-inflammatory activity with a range of 41.978 ± 1.634 to 93.194 ± 0.353% in RBC hemolysis inhibition (IC_50_ 4.956 μg/mL) and 38.260 ± 2.081 to 97.116 ± 0.679 in BSA denaturation inhibition (IC_50_ 3.216 μg/mL). Thus, similar efficiency for EAESTL as for a prominent anti-inflammatory drug, ibuprofen, can be noted.

### 3.5 EAESTL exhibited potential antibacterial efficacy against a number of microorganisms that cause disease in humans

**Fig. 3:**
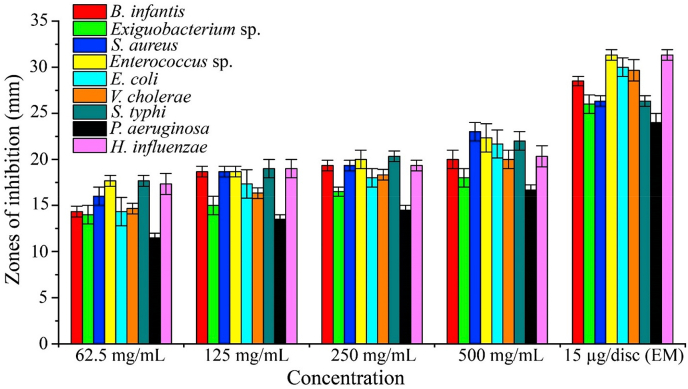
Antibacterial activity of EAESTL. Different concentrations of EAESTL and the commercial antibiotic erythromycin (EM) produced zones of inhibition against Gram-positive (*B. infantis*, *Exiguobacterium* sp., *S. aureus,* and *Enterococcus* sp.) and Gram-negative bacteria (*E. coli*, *V. cholerae*, *S. typhi*, *P. aeruginosa*, and *H. influenzae*). The proportions of inhibition zones were portrayed in millimeters with respect to the concentration. The data are represented by the mean ± STD (n = 3).

Medicinal plants containing bioactive phytochemicals have been used as a starting point for antibiotic synthesis to treat infectious diseases. Therefore, we assessed the antibacterial potential of EAESTL. Our findings revealed that EAESTL, at rates ranging from 62.5 to 500 mg/mL, can prevent the proliferation of bacteria, with inhibition zones ranging from 14 ± 1 to 23 ± 1 mm for Gram-positive bacteria and 11.5 ± 0.5 to 22 ± 1 mm for Gram-negative bacteria (Supplementary Figure S4). This finding demonstrates EAESTL's antibacterial effectiveness across a broad range of microorganisms. As shown in Fig. 3, against all the bacteria tested, a greater inhibition zone was obtained at a concentration of 500 mg/mL. EAESTL exhibited the highest antibacterial activity with a 23 ± 1 mm inhibition zone against *S. aureus* at 500 mg/mL, and the lowest antibacterial efficacy was 11.5 ± 0.5 mm against *P. aeruginosa* at 62.5 mg/mL. *P. aeruginosa* was always at the lowest level in terms of the zone of inhibition, while the growth of *S. aureus* was inhibited more than that of any other bacterial type studied. Erythromycin (EM) was used as a standard during this study and showed antibacterial activity with inhibition zones extending from 24 ± 1 to 31.333 ± 0.577 mm, which is comparable with EAESTL at a concentration of 500 mg/mL. The MIC and MBC values for the EAESTL tested on the nine bacterial strains are shown in Supplementary Table S1. The values recorded for MBC were greater than those for MIC, ranging from 3.270 ± 1.133 to 6.541 ± 2.266 mg/mL.

### 3.6 EAESTL has been found to be safe in cytotoxicity and acute toxicity tests

**Fig. 4:**
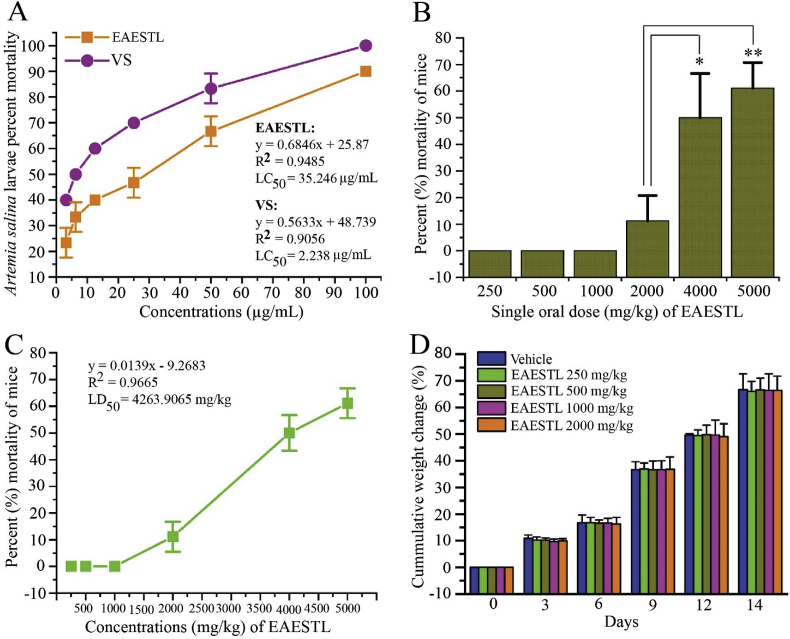
The toxicity of EAESTL. (A) EAESTL cytotoxicity in *Artemia salina* larvae. (B) The effect of EAESTL dosage on mouse mortality. (C) The median lethal dose (LD_50_) of EAESTL in acute toxicity. (D) Effects of acute EAESTL treatment on body weight.

The cytotoxicity of EAESTL was tested using an *Artemia salina* larvae lethality bioassay. In this study, all *Artemia salina* larvae survived under control conditions after 24 h of EAESTL treatment. The mortality of *Artemia salina* larvae was proportionally dependent on the concentration of EAESTL up to 24 h of treatment time. Here, 100% of *Artemia salina* larvae died at a concentration of 100 μg/mL vincristine sulfate, while 90% died at the same concentration of EAESTL. As shown in Fig. 4A, the cytotoxic effect (LC_50_) of EAESTL was 35.246 μg/mL; however, under the same conditions for a standard anticancer drug (vincristine sulfate), it was 2.238 μg/mL, and up to 100 μg/mL is considered nontoxic. Therefore, the cytotoxic effect of EAESTL can be considered moderately cytotoxic. The general focus of the acute toxicity studies was on determining the safe acute doses. Acute toxicity is generally expressed by the LD_50_ value, which denotes the amount of potential poisoning material given at once for a short-term (usually less than 24 h) interval that causes the death of 50% of a group of test animals. In this work, the LD_50_ was determined for Swiss albino mice. Any behavioral changes were also monitored to identify additional symptoms. None of the mice died after 24 h (Fig. 4B); however, after 40–43 h of dose implementation, 11.11 ± 9.622%, 50 ± 16.666%, and 61.111 ± 9.622% of the mice died at doses of 2000, 4000, and 5000 mg/kg b. w., respectively. Mouse mortalities that appeared within 40–43 h and up to 14 days of EAESTL (>2000 mg/kg) administration were considered for computing the oral LD_50_. The EAESTL oral LD_50_ value for the mice was 4263.906 mg/kg b. w. (Fig. 4C), which is more than two times higher than the nontoxic limit (>2,000 mg/kg b. w.) [83] and can be employed for further evaluation as a therapeutic agent to examine the effect of EAESTL on life-related parameters. During a 14-day observation period, EAESTL at a dose of 2000 mg/kg b. w. caused no death, behavioral abnormalities, locomotor ataxia, or diarrhea. At a dose of 2000 mg/kg b. w. (Fig. 4D), no noteworthy effect on body weight was noticed, and weight loss did not appear with increasing doses. From the behavioral monitoring, the food intake or water consumption attitude was similar for both test and control mice. This result indicates that EAESTL does not change the expected behavior of participating animals.

### 3.7 Sixteen phytochemicals exhibited good ADME to be possible drug candidates

The ADME profiles of 51 phytochemicals that showed individual antioxidant, anti-inflammatory, and antibacterial potential in the PASS program were investigated (Table 3). The bioavailability of a drug is greatly affected by the route of drug administration, such as human oral bioavailability (HOB) and human intestinal absorption (HIA). Therefore, HIA and HOB were calculated at different drug development stages. Among the 51 phytochemicals tested, 50 showed positive HIA, and 34 exhibited good HOB. In the gastrointestinal tract, P-glycoproteins (Pgp) affect drug absorption. Our findings demonstrated that 48 phytochemicals are noninhibitors and 51 phytochemicals are nonsubstrates of Pgp. Thus, the pharmacokinetic efficacy of phytochemicals is neither inhibited nor lowered by Pgp. Some medications pass through the BBB, while others do not. All of the tested phytochemicals (51) showed no BBB permeability, indicating non-CNS targets. The impact of plasma proteins on drug delivery is the largest. Only the free (unbound to plasma proteins) drug can reach the tissues and produce a therapeutic benefit. Drugs with limited PPB (<90%) are thought to be therapeutically effective. We determined that 23 out of 51 phytochemicals have optimal plasma protein-binding potential with less than 90% PPB. Drug metabolism reduces the bioavailability of a drug. The endoplasmic reticulum of the liver pertains to a group of enzymes called cytochrome P450 (CYP450) that are needed for xenobiotic detoxification. Most orally administered drugs were also metabolized by these enzymes. Drugs that repress CYPs can be hazardous because of the aggregation of coadministered medications or the actual medicine. We observed that 51 phytochemicals are noninhibitors of CYP4503A4, 45 are nonsubstrates of CYP4503A4, 51 are nonsubstrates of CYP4502D6, 48 are nonsubstrates of CYP4502D6, 51 are nonsubstrates of CYP4502C9 and 47 are nonsubstrates of CYP4502C9, indicating that these phytochemicals neither accumulate nor undergo metabolism in the liver and will be able to enter the systemic circulation for distribution throughout the body to exert their desired action. Pharmacokinetics also manifest how drugs reach their target sites of action and are excreted. One of the most important routes for eliminating a metabolized and unaltered chemical is through renal excretion. Substances that will become future drug-like compounds must have excretion qualities during the drug development process. We revealed that 30 phytochemicals have modest clearance potential and that 2 phytochemicals have a high clearance tendency. However, above all, among 51 phytochemicals, 16 phytochemicals (PN3, PN6, PN11, PN12, PN13, PN14, PN17, PN18, PN23, PN25, PN28, PN32, PN35, PN37, PN42 and PN43) showed good ADME.

**Table 3:** The ADME profiles of the 51 phytochemicals that showed antioxidant, anti-inflammatory, and antibacterial activities in the PASS program.

Peak No.	Compounds name	Compound CID	Canonical SMILES	Absorption				Distribution		Metabolism						Excretion
										CYP450 inhibitor			CYP450 substrate			
				HIA	HOB	p-glycoprotein inhibitor	p-glycoprotein substrate	BBB penetration	PPB (%)	CYP4502C9	CYP4502D6	CYP4503A4	CYP4502C9	CYP4502D6	CYP4503A4	Renal clearance (ml/min/kg)
3	1,1-Cyclohexanedimethanol	250594	C1CCC(CC1)(CO)CO	+	+	NI	NS	−	19.524	NI	NI	NI	NS	NS	NS	6.556 (Moderate)
6	4-Cyclopentene-1,3-dione	70258	C1C(=O)C]CC1=O	+	+	NI	NS	−	57.697	NI	NI	NI	NS	NS	NS	8.913 (Moderate)
11	O-Xylene	7237	CC1=CC]CC]C1C	+	+	NI	NS	−	89.002	NI	NI	NI	NS	NS	NS	10.483 (Moderate)
12	1,3,5,7-Cyclooctatetraene	637866	C1=CC]CC]CC]C1	+	+	NI	NS	−	84.437	NI	NI	NI	NS	NS	NS	2.413 (Low)
13	2-Furanone	10341	C1C]CC(=O)O1	+	+	NI	NS	−	79.219	NI	NI	NI	NS	NS	NS	14.220 (Moderate)
14	2,6,6-Trimethylbicyclo [3.1.1] heptane-3-ol or Isopinocampheol or 3-Pinanol	99038	CC1C2CC(C2(C)C)CC1O	+	+	NI	NS	−	54.637	NI	NI	NI	NS	NS	NS	15.208 (High)
17	5-Methylfuran-2-carbaldehyde or 5-Methyl furfural	12097	CC1=CC]C(O1)C]O	+	+	NI	NS	−	70.804	NI	NI	NI	NS	NS	NS	6.582 (Moderate)
18	3-Hydroperoxyhexane or 3-Hexyl hydroperoxide	141085	CCCC(CC)OO	+	+	NI	NS	−	59.289	NI	NI	NI	NS	NS	NS	8.870 (Moderate)
23	3-Methoxy-2- (methoxymethyl)-2-methylpropan-1-ol	542357	CC(CO)(COC)COC	+	+	NI	NS	−	11.982	NI	NI	NI	NS	NS	NS	5.628 (Moderate)
25	5-(2-Ethyl-1,3,2-dioxaborolan-4-yl)-3,4-dihydroxyfuran-2(5H)-one	54685836	B1(OCC(O1)C2C(=C(C(=O)O2)O)O)CC	+	+	NI	NS	−	85.147	NI	NI	NI	NS	NS	NS	11.526 (Moderate)
28	Benzyl alcohol	244	C1=CC]C(C]C1)CO	+	+	NI	NS	−	51.396	NI	NI	NI	NS	NS	NS	9.037 (Moderate)
32	2,3,4,6,7,8-Hexahydropyrrolo[1,2-a]pyrimidine	76349	C1CC2 = NCCCN2C1	+	+	NI	NS	−	45.148	NI	NI	NI	NS	NS	NS	12.065 (Moderate)
35	3-(Hydroxy-phenyl-methyl)-2,3-dimethyl-octan-4-one	559104	CCCCC(=O)C(C)(C(C)C)C(C1=CC]CC]C1)O	+	+	NI	NS	−	91.190	NI	NI	NI	NS	NS	NS	10.550 (Moderate)
37	Phenylethyl alcohol	6054	C1=CC]C(C]C1)CCO	+	+	NI	NS	−	53.125	NI	NI	NI	NS	S	NS	10.085 (Moderate)
42	5-(Hydroxymethyl)furan-2-carbaldehyde or 5-hydroxymethylfurfural	237332	C1=C(OC(=C1)C]O)CO	+	+	NI	NS	−	50.357	NI	NI	NI	NS	NS	NS	7.185 (Moderate)
43	Resorcinol	5054	C1=CC(=CC(=C1)O)O	+	+	NI	NS	−	44.189	NI	NI	NI	NS	NS	NS	16.027 (High)

Here, “+” and “−” means present and absent, respectively; I = Inhibitor; NI = Noninhibitor; NS = Non-substrate; S = Substrate.

### 3.8 The drug-able properties of phytochemicals

A comprehensive evaluation of drug-able properties and toxicity is required for effective drug development. These properties greatly influence the ADME of the drug candidates. In this study, the SwissADME server was used to quantify these properties (Table 4) for the identified phytochemicals of EAESTL. Lipinski suggested RO5, which states that the four basic physicochemical properties are MW (<500 g/mol), HBA ≤ 10, HBD ≤ 5, cLogP 1> and ≤5. These are all correlated with higher HOB. Our computational study for EAESTL found that 49 phytochemicals have an optimal MW (<500 g/mol), and all 51 phytochemicals have an appropriate number of HBA (≤10) and HBD (≤5). In addition, 27 phytochemicals have a standard assortment of cLogP (1> and <5). The ideal RB range is 0–11, while the highest border for effective absorption is 13. Forty-four phytochemicals contain RB attributed to the standard range. Compound absorption is also connected to TPSA, and a TPSA of ≤140 is associated with high compound absorption. All identified phytochemicals have a TPSA value of <106.97, indicating that they have high permeability. The LogS value, which is always preferably low, impacts drug solubility. Twenty-eight phytochemicals have a suitable LogS value range of −4.0 to 0.5, indicating that they can cross the membrane bilayer. Toxicity prediction is essential in the current drug discovery process for assessing a compound's adverse effects on an animal and the environment. The hERG gene (human ether-a-go-go-related gene) generally codes for a potential-dependent potassium ion channel that controls heartbeat. Many drugs have been abandoned due to cardiotoxicity induced by hERG inhibition. Among the 51 phytochemicals, 49 are noninhibitors of hERG. An obstacle to medicinal agents is carcinogenicity, which becomes severe when linked with mutagenicity. The Ames test is the most popular way of determining mutagenicity. Forty-five phytochemicals of EAESTL were found to be nonmutagenic in an Ames test (Table 4). Liver toxicity is one of the most critical aspects of the medication development process. The hepatotoxicity test revealed that 50 phytochemicals are not hepatotoxic. Acute oral toxicity is an essential endpoint in medicinal research and environmental risk management. However, the experiments are tedious to conduct; therefore, *in silico* approaches have been developed as a replacement. The United States Environmental Protection Agency (EPA) has classified toxicity based on LD_50_, and we found that among 51 phytochemicals, 42 are not acutely toxic. One of the well-known methods of evaluating a drug's drug-likeness is RO5. In this study, the SwissADME server was used to assess the compounds' RO5, and 36 phytochemicals did not violate this set of rules, indicating that they are drug-like. The synthetic accessibility of a drug candidate is an important factor; for this reason, computational and medicinal chemists consider this to be a basic objective in the early drug development stage. Compounds with a synthetic accessibility score (SAscore) ≥6 are challenging to synthesize, and those with an SAscore ≤6 are easy to synthesize. Forty-eight phytochemicals showed a favorable synthetic accessibility score of ≤6 and the desired properties. However, only eight phytochemicals (PN7, PN14, PN24, PN28, PN34, PN35, PN37 and PN46) exhibited optimal druggable properties without toxicity. Taken together, four phytochemicals (PN14, PN28, PN35, and PN37) passed ADME and demonstrated optimal druggable properties without toxicity.

**Table 4:** The drug-able properties (physicochemical, lipophilicity, water solubility, drug-likeness (RO5) and synthetic accessibility properties) and toxicity of 51 phytochemicals.

Peak No.	Compound CID	Canonical SMILES	Physicochemical properties					Lipophilicity (cLOGPo/w)	Water solubility [LogS (ESOL)]	Drug likeness	Medicinal chemistry	Toxicity				
			MW (g/mol)	No. of HBA	No. of HBD	No. of rotatable bonds	TPSA			RO5 violation	Synthetic accessibility score	hERG inhibitor	AMES toxicity	Carcinogenicity	Hepatotoxicity	Rat oral acute toxicity
7	91698641	CCOCCOC(=O)CC(C)C	174.24	3	0	7	35.53	1.86	−1.47	0	2.09	NI	NT	NC	NT	NT
14	99038	CC1C2CC(C2(C)C)CC1O	154.25	1	1	0	20.23	2.25	−2.4	0	3.8	NI	NT	NC	NT	NT
24	98219	CCCCCCCC(C)Br	207.15	0	0	6	0	3.98	−3.81	0	3.91	NI	NT	NC	NT	NT
28	244	C1=CC]C(C]C1)CO	108.14	1	1	1	20.23	1.41	−1.69	0	1	NI	NT	NC	NT	NT
34	31276	CC(C)CCOC(=O)C	130.18	2	0	4	26.3	1.76	−1.8	0	1.19	NI	NT	NC	NT	NT
35	559104	CCCCC(=O)C(C)(C(C)C)C(C1=CC]CC]C1)O	262.39	2	1	7	37.3	3.66	−3.65	0	2.74	NI	NT	NC	NT	NT
37	6054	C1=CC]C(C]C1)CCO	122.16	1	1	2	20.23	1.64	−1.82	0	1	NI	NT	NC	NT	NT
46	24847	CCCCCC(CCC)CO	158.28	1	1	7	20.23	3.08	−2.72	0	2.1	NI	NT	NC	NT	NT

I = Inhibitor; NI = Non-inhibitor; NC = Noncarcinognic; NT = Non-toxic; T = Toxic.

## 4 Discussion

Plants generate secondary metabolites and utilize them to defend themselves against possible adversaries and environmental risks. These metabolites and their derivatives have been effective therapeutic agents from the beginning of human evolution and play a significant role in treating various illnesses [84]. Therefore, this study explored the *in vitro* antioxidant, anti-inflammatory, and antibacterial activities of EAESTL and identified the phytochemicals in the extract to discover phytochemical compounds by means of a computational study of pharmacokinetics (ADME), toxicity, drug-able properties, and the probability of biological activities and *in vivo* cytotoxicity and acute toxicity to aid in developing potential therapeutic agents against oxidants, inflammation, and bacterial infection.

In living organisms, ROS are created endogenously or exogenously. The external supply of antioxidants acts as either ROS scavengers or inducers of endogenous (enzymatic and nonenzymatic) antioxidative systems to fight against ROS. Natural antioxidants produced from medicinal and dietary plants are commonly used as therapeutic agents. Therefore, we determined the antioxidant activity of EAESTL by using DPPH free radical- and H_2_O_2_-scavenging activity methods. EAESTL solution converted purple-colored DPPH to a yellow-colored DPPH-H protonated substance. Thus, the absorbance of DPPH diminished with increasing EAESTL condensation, indicating the free radical-scavenging potency of EAESTL. Similar free radical-scavenging activity was reported for the ethyl acetate extract of *Xylocarpus granatum* leaves by Darmadi et al. [85]. H_2_O_2_ is not a free radical but it is an electrically neutral ROS. It can generate a highly reactive hydroxyl radical, which severely damages adjacent biomolecules [86]. In addition, it is hazardous due to its mobility, typically passing through cellular membranes and arriving at the cell compartments far from the site of its origination [87]. Thus, the removal of H_2_O_2_ is crucial to avoid oxidative damage. Our antioxidant activity results showed that EAESTL scavenges H_2_O_2_ in a dose-related manner, which is consistent with the known antioxidant capacities of the ethyl acetate extract of *Fructus ligustri lucidi* [88] and the methanol extract of *Glinus oppositifolius* [89]. The DPPH and H_2_O_2_ scavenging capacity of EAESTL may arise from the phenolics, terpenoids, vitamin C, vitamin E, and carotene in EAESTL.

During inflammation, leukocytes discharge lysosomal enzymes, and proteases are part of their protective roles, causing more damage to the tissue [90]. Preventing hemolysis of the RBC membrane can provide insight into anti-inflammation because the RBC membrane and the lysosome membrane are identical. The firmness of such a plasma membrane can prevent or postpone cytolysis, preventing the liberation of cytosolic elements and, consequently, preventing tissue injury and the inflammatory reaction. Protein denaturation is one additional reason for inflammation [91]. An aid that can inhibit protein damage could be helpful in the treatment of inflammatory disorders. The ability of EAESTL to prevent RBC lysis and BSA protein denaturation was assessed as part of the research into the process of treating symptoms of inflammation. EAESTL exhibited concentration-dependent anti-inflammatory activity by inhibiting RBC lysis and BSA protein denaturation. The results demonstrate that this anti-inflammatory effect is perhaps attributed to the presence of esters, phenolics, terpenes, hydrocarbons, ketones, and aldehydes in EAESTL. Similar anti-inflammatory activity has also been observed in the research conducted by Gunathilake et al. [29], which showed that extracts of *Cassia auriculata*, *Passiflora edulis*, *Sesbania grandiflora*, *Olax zeylanica*, *Gymnema lactiferum*, and *Centella asiatica* prevented RBC lysis and BSA protein denaturation.

The bacterial strains used in this study are infectious in nature and can induce diseases. For instance, *B. infantis* was found in a neonate with sepsis, and *Exiguobacterium* sp. promotes pneumonia (CAP) and bacteremia [92,93]. *S. aureus* is among the most prevalent human pathogens, exacerbating bacteremia, respiratory tract and soft tissue infections, and endocarditis [94]. Some *Streptococcus* sp. involved in the pathogenesis of upper respiratory infections [95], and virulent *E. coli* accelerate gastroenteritis and urinary tract infections [96]. *V. cholerae* is an enteric pathogen that releases cholera toxin and causes acute secretory diarrhea [97]. *S. typhi* is an MDR-resistant bacterial pathogen that usually causes typhoid and paratyphoid, and it has rapidly gained resistance to previously efficacious drugs, such as ciprofloxacin [98]. *P. aeruginosa* can induce significant life-threatening infections by preventing the immune systems of affected individuals [99]. *H. influenzae* is the main bacterial agent that spreads diseases in the respiratory and sensory systems of the body [100]. Therefore, the antibiotic activity of EAESTL was assessed against these bacteria. The presence of physiologically active chemicals such as phenolics, esters, terpenes, hydrocarbons, aldehydes, and ketones could account for this antibacterial action. Our findings are in accordance with the previous research data that plant extracts high in phenolic compounds [101], fatty acid methyl esters [102], terpenes, hydrocarbons [103], aldehydes, and ketones [104] have antibacterial activity. Our recent observations show that the ethyl acetate extract of *Ruellia prostrata* Poir. has significant antibacterial activity against *B. infantis*, *Exiguobacterium* sp., *E. coli,* and *P. aeruginosa* [24], suggesting a similar potential for inhibiting these pathogenic bacteria that share a common carnage mechanism. The study conducted by Saranraj et al. [105], in accordance with our results, found that an ethyl acetate extract of *Acalypha indica* plant leaves had antibacterial activity against bacterial strains of S. *aureus*, *S. typhi*, *V. cholerae*, and *E. coli*.

The unexpected side effects of a particular drug compound can be avoided by estimating the level of toxicity with respect to the dose. Therefore, the cytotoxicity and acute oral toxicity studies were performed in *Artemia salina* larvae and mice, and we found that the LC_50_ and LD_50_ of EAESTL were 35.246 μg/mL and 4263.906 mg/kg, respectively. The LC_50_ value of EAESTL is lower than the LC_50_ value of standard vincristine sulfate. The LD_50_ value of EAESTL is beyond 2,000 mg/kg, which indicates that it is nontoxic. In the acute toxicity study, if the LD_50_ value is above 2000 mg/kg, it is considered nontoxic [83]. Thereby, EAESTL can be a good source of potential therapeutic agents. Successful drug development entails an accurate evaluation and rationalization of pharmacokinetics (ADME) and drug-able properties. Therefore, we investigated these properties *in silico* and discovered that only four phytochemicals, namely, 2,6,6-trimethylbicyclo [3.1.1] heptane-3-ol (PN14), benzyl alcohol (PN28), 3-(hydroxy-phenyl-methyl)-2,3-dimethyloctan-4-one (PN35), and phenylethyl alcohol (PN37), passed ADME and demonstrated good drug-able properties without toxicity. Among these four phytochemicals, 2,6,6-trimethylbicyclo [3.1.1] heptane-3-ol also known as isopinocampheol or 3-pinanol, a terpenoid in nature, was found to have anti-inflammatory and antibacterial activities. In accordance with our results, *in vitro* activity investigations of essential oils from *Hyssopus officinalis* [51], *Cedrelopsis grevei* leaves [106] and the hexane-ether extract of *Tanacetum santolinoides* [107] have been reported to contain 2,6,6-trimethylbicyclo [3.1.1] heptane-3-ol, which has anti-inflammatory and antimicrobial properties. Benzyl alcohol exhibited anti-inflammatory activity. Consistent with our findings, the chloroform fraction of aerial parts of *Isodon rugosus* Wall. ex Benth contains benzyl alcohol along with other bioactive phytochemicals that have been reported to have anti-inflammatory and analgesic activity when analyzed *in vivo* [58]. Both the ethanol extract of *Albizia coriaria* (Welw ex. Oliver) leaves and the ethyl acetate extract of *Albizia coriaria* (Welw ex. Oliver) stem bark contain benzyl alcohol as a significant component and are reported to have *in vitro* antioxidant and antibacterial properties [59]. 3-(hydroxy-phenyl-methyl)-2,3-dimethyl-octan-4-one was found to have anti-inflammatory activity. Consistently, an ethyl acetate extract of *Ruellia prostrata* Poir. aerial parts contains 3-(hydroxy-phenyl-methyl)-2,3-dimethyl-octan-4-one, a phenolic molecule with anti-inflammatory properties [24]. Phenylethyl alcohol displayed anti-inflammatory activity. Our findings are also in good agreement with the results reported by Dadkhah et al. [59] and Amor et al. [63] who observed that phenylethyl alcohol is one of the main phytoconstituents of *Rosa damascena* Mill. and *Phlomis* species essential oils and exhibited antioxidant and anti-inflammatory activities, respectively. In addition, phenylethyl alcohol is a driven compound of *Trichoderma virens* 7b hexane extract, which showed antifungal activity against *Ganoderma boninense*, the causal agent of a devastating disease affecting oil palm in Southeast Asian countries [60]. Collectively, our results may contribute to the development of antioxidant, anti-inflammatory, and antibacterial agents for humans. Moreover, this work also shows a correlation between the ethnomedicinal uses of *Senna tora* (L.) Roxb leaves and their pharmacological activities.

## 5 Conclusion

The findings of this study concluded that *Senna tora* (L.) Roxb. leaves contain antioxidant, anti-inflammatory, and antibacterial activities due to the presence of some biologically active phytochemicals, viz., phenolics, terpenoids, vitamin C, vitamin E, carotene, esters, hydrocarbons, ketones, and aldehydes. Based on pharmacokinetics, drug-able properties, and toxicity, this study also identified four phytochemicals including 2,6,6-trimethylbicyclo [3.1.1] heptane-3-ol, benzyl alcohol, 3-(hydroxy-phenyl-methyl)-2,3-dimethyl-octan-4-one, and phenylethyl alcohol, as possible therapeutic candidates. Among the four phytochemical compounds, 2,6,6-trimethylbicyclo [3.1.1] heptane-3-ol is anti-inflammatory and antibacterial; benzyl alcohol is an antioxidant, anti-inflammatory, and antibacterial; 3-(hydroxy-phenyl-methyl)-2,3-dimethyl-octan-4-one is anti-inflammatory; and phenylethyl alcohol is both an antioxidant and an anti-inflammatory. Further research is needed for the isolation of these phytochemical compounds with fascinating *in vitro* and *in vivo* pharmacological properties and to assess their safety and bioavailability in *in vivo* animal models to aid in developing drugs against oxidants, inflammation, and bacterial infection.

## Author contribution statement

Md. Mashiar Rahman and Shahina Akhter conceived and designed the experiments, contributed reagents, materials, analysis tools, or data, and wrote the paper. Md. Abdullah Al Noman, Shapla Khatun, Rahat Alam, Md. Mahade Hasan Shetu, Enamul Kabir Talukder, Raihan Rahman Imon, and Md. Yaman Biswas performed the experiments, analyzed and interpreted the data. K. M. Anis-Ul-Haque and Mohammad Jashim Uddin analyzed and interpreted the data and wrote the paper.

## Funding

No external funding was received for this research work.

## Declaration of competing interest

None.

## Abbreviations

DPPH: 2,2-diphenyl-1-picrylhydrazyl
BSA: Bovine Serum Albumin
RBC: Red Blood Cell
EAEST: Ethyl acetate extract of *Senna tora* (L.) Roxb. Leaves
PASS: Prediction of Activity Spectra for Substances
ADME: Absorption, Distribution, Metabolism, and Excretion
PN: Peak Number
NSAIDs: Nonsteroidal anti-inflammatory drugs
COX-1: Cyclooxygenase 1
COX-2: Cyclooxygenase 2
EGCG: Epigallocatechin-3-gallate
MeOH: Methanol
DMSO: Dimethyl Sulfoxide
PBS: Phosphate-Buffered Saline
OECD: Organization for Economic Co-operation and Development
GC-MS: Gas Chromatography Mass Spectrometry
hERG: Human Ether-a-go-go-Related gene
MW: Molecular Weight
HBA: Hydrogen Bond Acceptor
HBD: Hydrogen Bond Donor
RTB: Rotatable Bond
TPSA: Topological Polar Surface Area
PPB: Plasma Protein Binding
BBB: Blood Brain Barrier
HIA: Human Intestinal Absorption
HOB: Human Oral Bioavailability
RO5: Lipinski's Rule of Five
CYP450: Cytochrome P450
LD_50_: Lethal Dose 50
IC_50_: Inhibitory Concentration 50
STD: Standard Deviation
EM: Erythromycin
Pgp: P-glycoproteins
CNS: Central Nervous System
EPA: Environmental Protection Agency
CAP: Community-Acquired pneumonia
GI: Gastrointestinal
MIC: Minimum Inhibitory Concentration
MBC: Minimum Bactericidal Concentration
