# Supplementary material for: Evaluation of Senna tora (L.) Roxb. leaves as source of bioactive molecules with antioxidant, anti-inflammatory and antibacterial potential
Source: Heliyon. 2023 Jan 19;9(1):e12855. doi: 10.1016/j.heliyon.2023.e12855 (PMC9898628; doi:10.1016/j.heliyon.2023.e12855)
Supplement: Multimedia component 1 [file mmc1.docx]

**Supplementary Material**

**Evaluation of *Senna tora* (L.) Roxb. leaves as source of bioactive molecules with antioxidant, anti-inflammatory and antibacterial potential**

^
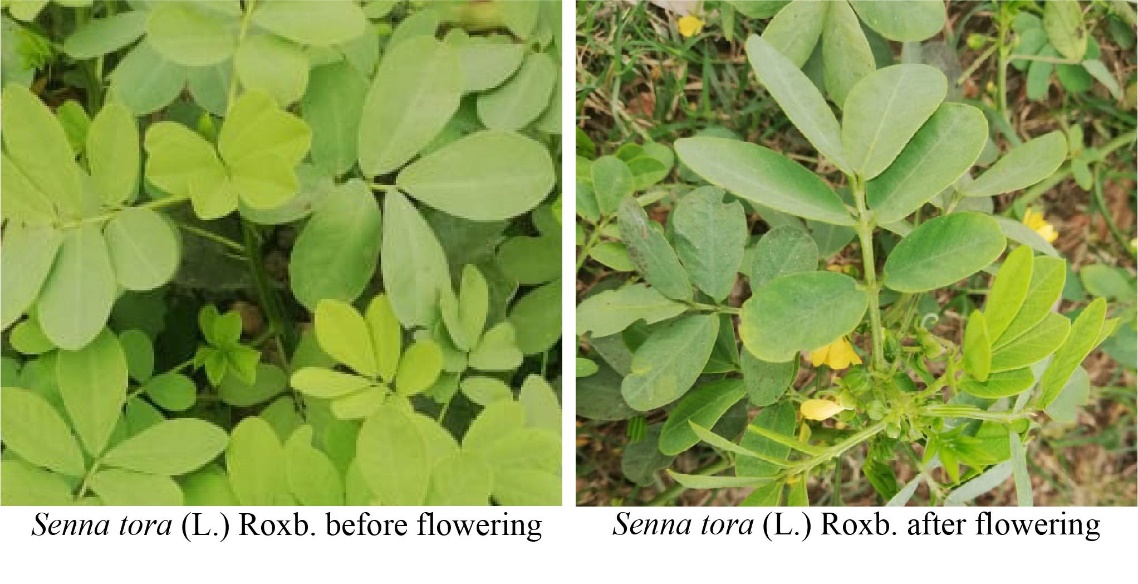
^

**Supplementary Figure S1.** Morphology of *Senna tora* (L.) Roxb. before flowering and after flowering.


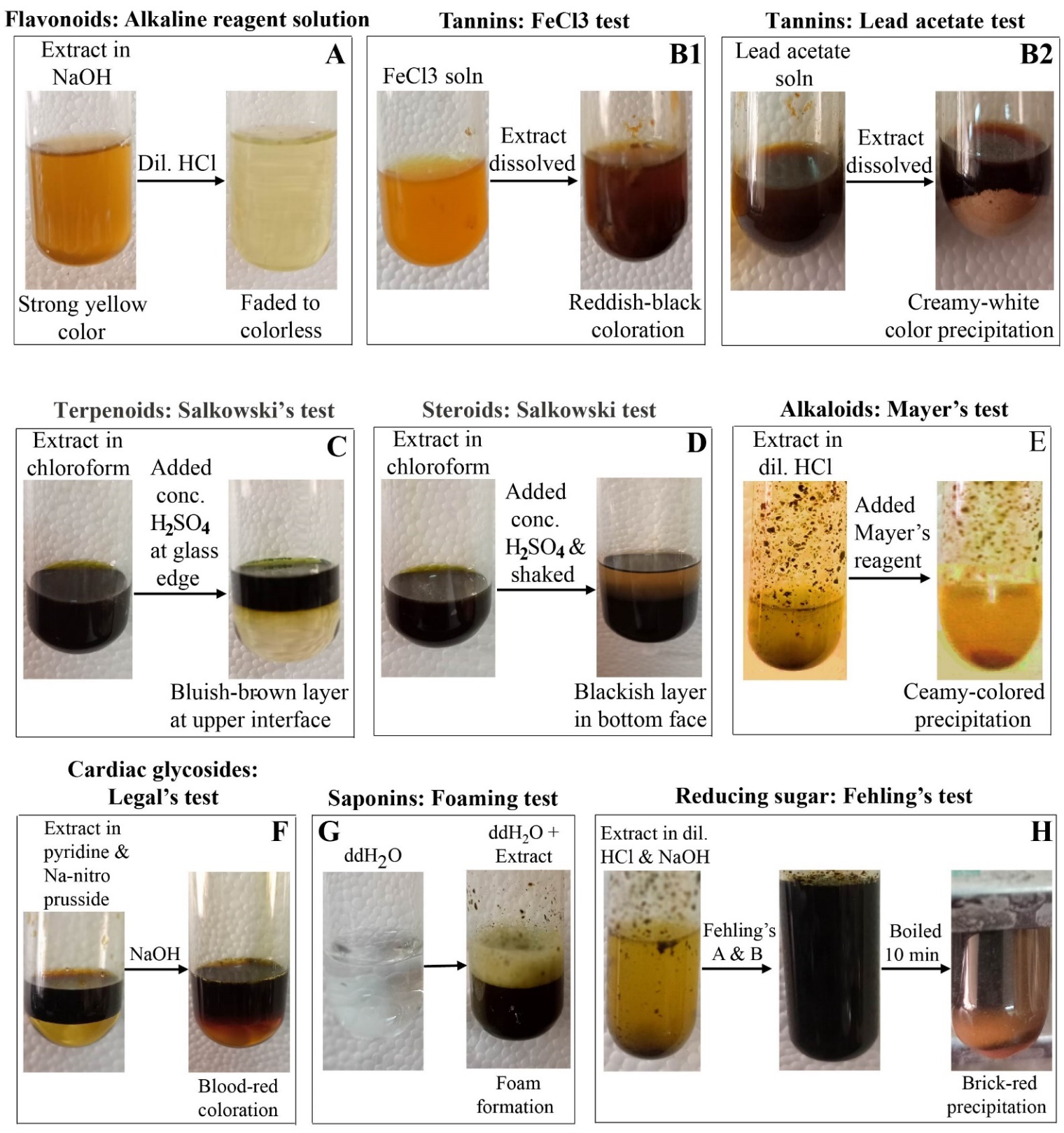


**Supplementary Figure S2.** Preliminary phytochemical screening of EAESTL by color change experiments. (A) Alkaline reagent test for flavonoids, (B1) FeCl3 and (B2) Lead acetate tests for tannins, (C) Salkowski’s test for terpenoids, (D) Salkowski’s test for steroids, (E) Mayer’s test for alkaloids, (F) Legal’s test for cardiac glycosides, (G) Foaming test for saponins and (H) Fehling’s test for reducing sugar.


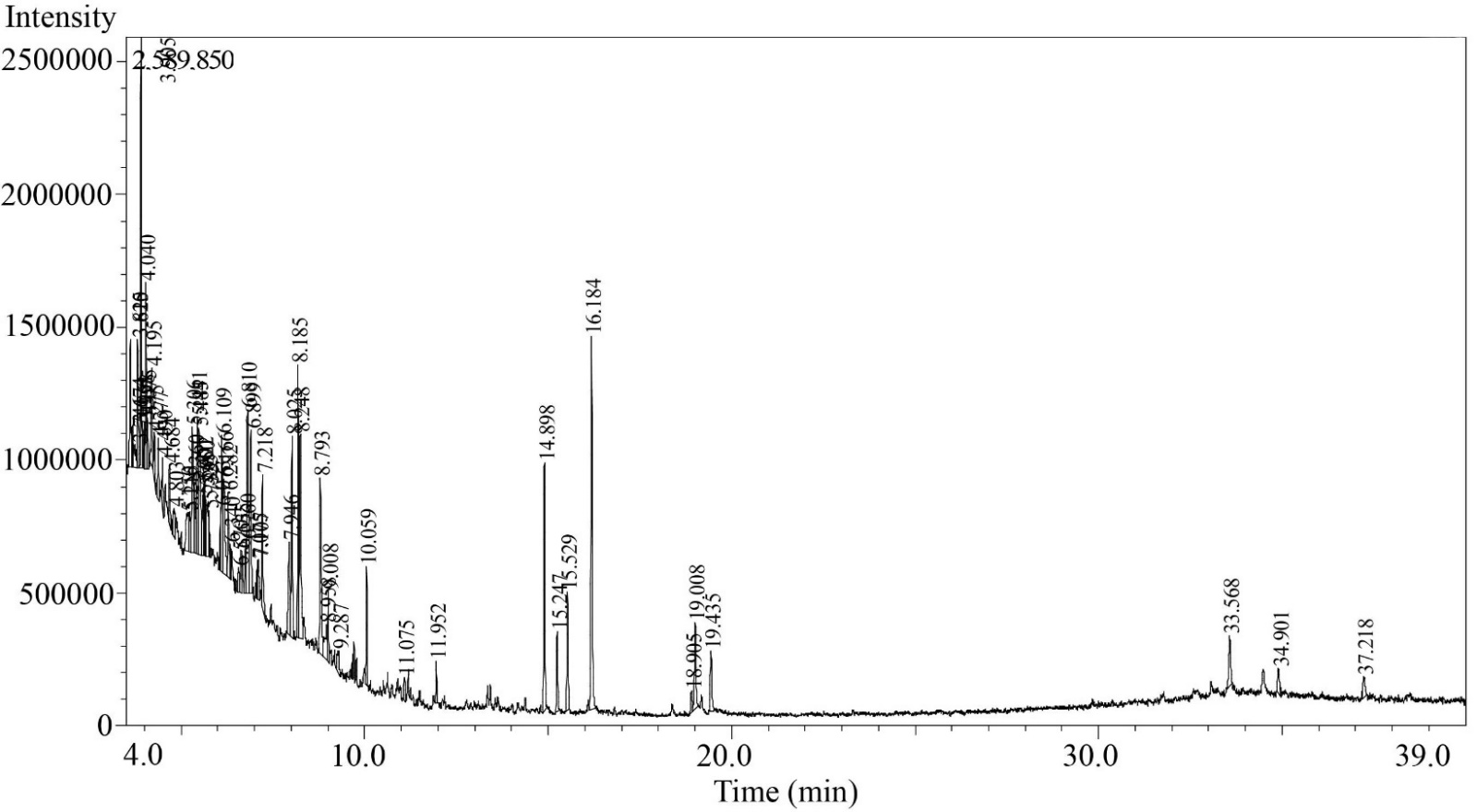


**Supplementary Figure S3.** GC-MS chromatogram of ethyl acetate extract of *Senna tora* (L.) Roxb. leaves (EAESTL).

**
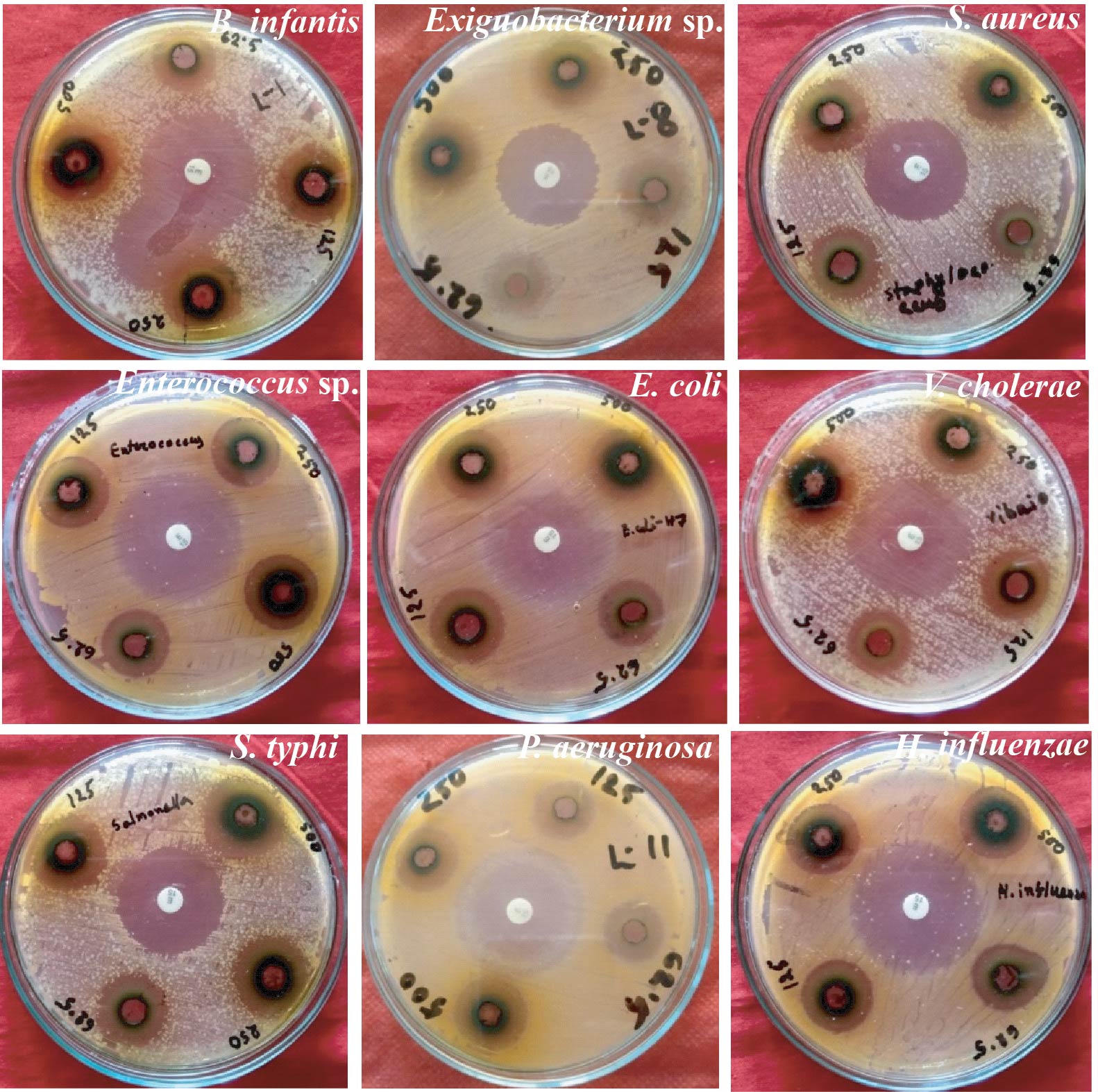
**

**Supplementary Figure S4.** Growth inhibition of some Gram-positive (*B. infantis*, *Exiguobacterium* sp., *S. aureus,* and *Enterococcus* sp.) and Gram-negative bacteria (*E. coli*, *V. cholerae*, *S. typhi*, *P. aeruginosa*, and *H. influenzae*) bacterial strains caused by EAESTL. The discs of erythromycin in the center of the Petri plates are used as a positive control.

**Table S1.** Minimum inhibitory concentration (MIC) and minimum bactericidal concentration (MBC) of EAESTL after 24 h.

| **SN** | **Bacterial strains** | **MIC (mg/mL)** | **MBC (mg/mL)** |
| --- | --- | --- | --- |
| 1. | *Bacillus infantis* | 5.233 ± 2.266 | 6.541 ± 2.266 |
| 2. | *Exiguobacterium sp.* | 5.233 ± 2.266 | 6.541 ± 2.266 |
| 3. | *Staphylococcus aureus* | 2.616 ±1.133 | 3.270 ± 1.133 |
| 4. | *Enterococcus sp.* | 2.616 ± 1.133 | 3.270 ± 1.133 |
| 5. | *Escherichia coli* | 2.616 ± 1.133 | 3.27 ± 1.133 |
| 6. | *Vibrio cholerae* | 2.616 ± 1.133 | 3.270 ± 1.133 |
| 7. | *Salmonella typhi* | 2.616 ± 1.133 | 3.270 ± 1.133 |
| 8. | *Pseudomonas aeruginosa* | 5.233 ± 2.266 | 6.541 ± 2.266 |
| 9. | *Haemophilus influenzae* | 2.616 ± 1.133 | 3.270 ± 1.133 |

The MIC and MBC were performed three times. The values are the mean ± STDEV.
